# *In Vitro* Growth Inhibitory Activities of Natural Products from Irciniid Sponges against Cancer Cells: A Comparative Study

**DOI:** 10.1155/2016/5318176

**Published:** 2016-08-11

**Authors:** Yosr BenRedjem Romdhane, Monia Elbour, Marianna Carbone, Maria Letizia Ciavatta, Margherita Gavagnin, Véronique Mathieu, Florence Lefranc, Leila Ktari, Karim Ben Mustapha, Abdellatif Boudabous, Robert Kiss, Ernesto Mollo

**Affiliations:** ^1^Institut National des Sciences et Technologies de la Mer (INSTM), Salammbô, 2025 Tunis, Tunisia; ^2^Consiglio Nazionale delle Ricerche (CNR), Istituto di Chimica Biomolecolare (ICB), 80078 Pozzuoli, Italy; ^3^Laboratoire de Cancérologie et de Toxicologie Expérimentale, Faculté de Pharmacie, Université Libre de Bruxelles (ULB), 1050 Brussels, Belgium; ^4^Service de Neurochirurgie, Hôpital Erasme, ULB, 1070 Brussels, Belgium; ^5^Laboratoire des Microorganismes et Biomolécules actives, Faculté des Sciences de Tunis, 2092 Tunis, Tunisia

## Abstract

Marine sponges of the Irciniidae family contain both bioactive furanosesterterpene tetronic acids (FTAs) and prenylated hydroquinones (PHQs). Both classes of compounds are known for their anti-inflammatory, antioxidant, and antimicrobial properties and known to display growth inhibitory effects against various human tumor cell lines. However, the different experimental conditions of the reported* in vitro* bioassays, carried out on different cancer cell lines within separate studies, prevent realistic actual discrimination between the two classes of compounds from being carried out in terms of growth inhibitory effects. In the present work, a chemical investigation of irciniid sponges from Tunisian coasts led to the purification of three known FTAs and three known PHQs. The* in vitro* growth inhibitory properties of the six purified compounds have been evaluated in the same experiment in a panel of five human and one murine cancer cell lines displaying various levels of sensitivity to proapoptotic stimuli. Surprisingly, FTAs and PHQs elicited distinct profiles of growth inhibitory-responses, differing by one to two orders of magnitude in favor of the PHQs in all cell lines. The obtained comparative results are discussed in the light of a better selection of drug candidates from natural sources.

## 1. Introduction

The great majority of cancer patients (i.e., ~90%) die from their metastases because metastatic cancers are resistant to almost any type of currently available treatment [1, 2], while the survival rates of patients with metastatic or recurrent cancers have remained virtually unchanged during the past 30 years [2]. Cancer cells and especially metastatic cancer cells resist cytotoxic insults through multiple and biologically complex mechanisms including dual roles for autophagy [3], the so-called multidrug resistance (MDR) phenotype [4, 5], cancer cell dormancy [6, 7], cancer stem cells [8, 9], an extraordinary sophisticated tumor microenvironment [10–12], hypoxia [13], and also the resistance to proapoptotic stimuli [14, 15]. There is thus urgent need for new effective drugs in oncology that could face the extraordinary complex phenomenon of the metastatic process. In this area of research, the exploration of the nature chemical diversity still remains a major option to select the best candidates as novel therapeutics against metastatic cancers. In general, initial selection of promising bioactive products is made by evaluating their* in vitro* growth inhibitory activity.

Among the richest natural sources of candidates as potential anticancer agents, marine sponges (phylum Porifera) already provided a wide range of cytotoxic metabolites with peculiar chemical structures. This report, in particular, focuses on compounds isolated from sponges of the genera* Ircinia* and* Sarcotragus* (Demospongiae: Dictyoceratida: Irciniidae).

Two major classes of compounds from irciniid sponges have especially attracted the attention of marine natural product chemists and pharmacologists: (a) linear terpenes containing both a furan ring and the tetronic acid moiety (FTAs) and (b) hydroquinones with a terpenoid portion (PHQs). Although it is still not clear whether the compounds are of dietary origin, produced by microbial symbionts, or* de novo* biosynthesized by the sponges themselves, a panel of bioactivities with pharmaceutical potential, including cytotoxic, anti-inflammatory, antioxidant, and antimicrobial properties, have been attributed to both classes of compounds [16–26]. It is worth mentioning, however, that compounds belonging to the two different groups of compounds isolated from irciniid sponges have not yet been compared in the same study using the same experimental procedures for growth inhibition assessments against the same panel of cancer cell lines, thus preventing from establishing reliable differences between themselves, at least* in vitro*, in terms of potential anticancer effects.

The following is a report on our experiments aimed at potency discrimination between compounds belonging to both classes of compounds in terms of* in vitro* growth inhibition in a panel of six cancer cell lines with distinct levels of biological aggressiveness as translated by distinct levels of sensitivity to proapoptotic stimuli. The research is thus especially aimed at assessing at which extent the different chemical features of the isolated compounds affect their* in vitro* growth inhibitory activities and if the patterns of* in vitro* growth inhibition of these compounds are similar to the ones displayed by classical cytotoxic proapoptotic ones. In other words, we explored the ability of the metabolites to induce cell death pathways that are mechanistically distinct from apoptosis [27, 28].

We first isolated compounds** 1**–**6** (Table 1) from samples of* Sarcotragus fasciculatus* (Pallas, 1766),* Sarcotragus spinosulus* Schmidt, 1862, and* Sarcotragus foetidus *Schmidt, 1862, collected along Tunisian coasts. Subsequently, to evaluate the growth inhibitory activities of the purified natural products, we have tried to recapitulate one of the characteristics of metastatic cancer cells, that is, their ability to resist proapoptotic cytotoxic insults, while using established cancer cell lines developing alone in plastic flasks. We have thus chosen six cancer cell lines (five human and one murine ones) with distinct levels of sensitivity to proapoptotic stimuli to determine the* in vitro* growth inhibitory activity of compounds** 1**–**6**. The selected human MCF-7 mammary adenocarcinoma [29] and Hs683 oligodendroglioma [30, 31] cell lines display actual sensitivity to proapoptotic stimuli, as does also the mouse B16F10 melanoma model [32, 33]. The remaining three human cancer cell lines display various levels of resistance to proapoptotic stimuli and they included the A549 non-small-cell lung cancer (NSCLC) [34], the SKMEL-28 melanoma [33], and the U373 glioblastoma [30, 31]. The growth inhibitory effects induced by compounds** 1**–**6** on these six cancer cell lines were determined by means of the MTT colorimetric assay as detailed previously [29–35].

## 2. Materials and Methods

### 2.1. General Experimental Procedures

NMR experiments were recorded at ICB-NMR Service Centre on a DRX 600 MHz Bruker spectrometer equipped with a TXI CryoProbe*™*, on a Bruker Avance-400 spectrometer using an inverse probe fitted with a gradient along the *z*-axis, and on a Bruker DPX-300 spectrometer. The NMR spectra were acquired in CDCl_3_ and in CD_3_OD. ESIMS and HRESIMS spectra were measured on a Micromass Q-TOF Microspectrometer coupled with HPLC Waters Alliance 2695. The instrument was calibrated by using a PEG mixture from 200 to 1000 MW. Optical rotations were measured using a Jasco DIP 370 digital spectropolarimeter. Analytical and preparative TLC were performed on precoated silica gel plates (Merck Kieselgel 60 F254, 0.2 mm and 0.5 mm), with detection provided by UV light (254 nm) and by spraying with ceric sulfate (CeSO_4_) reagent followed by heating (120°C). Silica gel column chromatography was performed using Merck Kieselgel 60 powder whereas size-exclusion chromatography was achieved on Sephadex LH-20 column.

### 2.2. Sponge Material and Taxonomic Identification

Four samples of three irciniid sponge species were collected by using SCUBA diving at a depth of about 15 m from three different sites along the coast of Tunisia. In particular,* S. spinosulus *was collected from Tabarka (36°58′3.65′′N, 8°45′53.89′′E) and Monastir (35°46′25.00′′N, 10°50′20.35′′E) in November 2010,* S. fasciculatus* (Pallas, 1766) was collected from Monastir in November 2010, while* S. foetidus* was collected from Cap Zebib (37°15′35.38′′N, 10°04′46.05′′E) in June 2011. The samples were kept frozen at −20°C until the extraction process. The colors of the samples were blackish for* S. foetidus *and* S. spinosulus* and red-brown for* S. fasciculatus*, with white-to-beige interior. The specimens were irregularly massive with a subspherical shape. The texture was resistant to tearing or cutting, with a firm and compressible consistency. The ectosome was thick and coarsely conulose. For taxonomic identification, sponge samples were preserved in 70% alcohol. Preparations of skeletons followed the standard practice proposed by Rützler [36]. The classification used in this work was that proposed by Hooper and van Soest [37], with the amendments given in the World Porifera Database [38]. The skeleton of the different species was a system of primary and secondary fibers that consists of laminated primary and secondary fibers and comprises numerous and fine spongin filaments. The diameter of primary fiber, secondary fiber, and spongin were, respectively, 100 *μ*m, 30 *μ*m, and 5 *μ*m for* S. foetidus*; 80 *μ*m, 40 *μ*m, and 0.7 *μ*m for* S. fasciculatus*; and 70 *μ*m, 30 *μ*m, and 2 *μ*m for* S. spinosulus.*

### 2.3. Extraction and Purification

Each sponge sample was exhaustively extracted with acetone at room temperature. After evaporation of the solvent in vacuo, the remaining aqueous phases were separately partitioned between H_2_O and Et_2_O. The Et_2_O portions from each extraction were evaporated under reduced pressure affording the corresponding crude Et_2_O extracts.

A portion (2 g) of the Et_2_O extract (3 g) of* S. spinosulus* from Tabarka was first fractionated on Sephadex LH-20 column (CHCl_3_/MeOH, 1 : 1) to give a fraction containing ircinin 1 (**1**) and compound** 5** as main metabolites. This fraction (546.0 mg) was then purified on silica gel column eluted with a gradient solvent system ranging from 100% to 20% light petroleum ether in Et_2_O to give pure compound** 5** (33.8 mg) [39] and ircinin 1 [**1**, 53.1 mg; [*α*]_D_ + 27.5 (*c* = 6.7, CHCl_3_); and [*α*]_D_ + 16.5 (*c* = 6.7, MeOH)] [16, 40, 41].

The Et_2_O extract (846.6 mg) of* S. spinosulus* from Monastir was fractionated on silica gel column eluted with a gradient solvent system ranging from 100% to 20% of light petroleum ether in Et_2_O affording a fraction (57.4 mg) containing compound** 4**. An aliquot (15.2 mg) of this fraction was further purified on preparative TLC (light petroleum ether/Et_2_O, 1 : 1) obtaining 12 mg of pure compound** 4 **[39].

An aliquot (2 g) of Et_2_O extract (8 g) from* S. fasciculatus* collected off the coast of Monastir was first subjected to Sephadex LH-20 column (CHCl_3_/MeOH, 1 : 1) affording a major fraction (500 mg) which was purified on silica gel column eluted with a gradient solvent system ranging from 80% to 30% of light petroleum ether in Et_2_O. Two selected fractions (40 mg and 60 mg) from this column containing sarcotin A (**2**) [16, 42] and variabilin (**3**) [43, 44], respectively, were further subjected to preparative TLC purification to give pure** 2** [15.5 mg; [*α*]_D_ + 28.0 (*c* = 7.7, CHCl_3_); and [*α*]_D_ + 17.5 (*c* = 7.7, MeOH)] and** 3** [21.3 mg; [*α*]_D_ − 21.5 (*c* = 1.5, CHCl_3_); and [*α*]_D_ − 10.5 (*c* = 1.5, MeOH)].

The Et_2_O extract (61.6 mg) from* S. foetidus *was subjected to silica gel column eluted with a gradient solvent system ranging from 100% to 0% light petroleum ether in diethyl ether affording pure compound** 6** (4.6 mg) [26].

All isolated compounds were identified by comparison of their spectral data (see Supplementary Material, available online at http://dx.doi.org/10.1155/2016/5318176) with the literature values [16, 26, 39–44].

### 2.4. Determination of* In Vitro* Anticancer Activity

The cell lines that we used for the MTT colorimetric assay (Table 2) are five human and one murine cancer cell lines with the following histological types and origins. The human cancer cell lines include the A549 NSCLC (DSMZ code ACC107), the Hs683 oligodendroglioma (American Type Culture Collection (ATCC) code HTB-138), the MCF-7 breast adenocarcinoma (Deutsche Sammlung von Mikroorganismen und Zellkulturen (DSMZ) code ACC115), the SKMEL-28 melanoma (ATCC code HTB-72), and the U373 glioblastoma (European Collection of Cell Culture (ECACC) code 08061901) cell lines. The B16F10 mouse melanoma cell line (ATCC code CRL-6475) was obtained from the ATCC collection (Manassas, VA).

The cells were cultured in RPMI (Lonza, Verviers, Belgium) medium supplemented with 10% heat inactivated foetal calf serum (Lonza). All culture media were supplemented with 4 mM glutamine, 100 *μ*g/mL gentamicin, and 200 U/mL penicillin and 200 *μ*g/mL streptomycin (Lonza). The overall growth level of the human cancer cell lines was determined using a colorimetric MTT (3-[4,5-dimethylthiazol-2yl]-diphenyl tetrazolium bromide, Sigma, Belgium) assay as detailed previously [29–32]. Compounds** 1**–**6** were dissolved in DMSO and redissolved in the cell culture media at a final concentration of 0.1%, which induces no observable toxic effects on cells. Six replicates of each experimental condition were performed. Thus, this procedure enables the concentration of compounds** 1**–**6** that decreased by 50% the growth of each cell line (GI_50_ concentration) after having cultured it with the compound of interest for 72 h (the GI_50_ index in *μ*M) to be determined.

## 3. Results

The chemical study of irciniid sponges collected along Tunisian coasts led to the isolation of the known FTAs ircinin 1 (**1**), sarcotin A (**2**), and variabilin (**3**), along with the known PHQs** 4**–**6** (Table 1). The isolated compounds were identified by comparison with ^1^H-NMR and ^13^C-NMR, mass spectrometry, and optical rotation data available in the literature [16, 26, 39–44].

Subsequently, we tested the* in vitro* growth inhibitory activities of the isolated natural products from irciniid sponges against six cancer cell lines and the obtained data are illustrated in Table 2.

All PHQs showed GI_50_ concentration <10 *μ*M on MCF-7, SKMEL-28, and B16F10 cell lines, while just compound** 6** displayed a homogeneous inhibitory pattern <10 *μ*M on all 6 cell lines tested. Conversely, the three FTAs showed higher GI_50_ concentration, always >60 *μ*M.

## 4. Discussion

Before proceeding to an interpretation of the data, we need to define when the compounds can be considered remarkably active with respect to the effects they produce on cancer cells. The GI_50_-related micromolar concentrations that define precisely the limits between “weakly active,” “active,” and “highly active” compounds in terms of growth inhibition are not following standardized rules in the literature. The instructions for authors of the Journal of Natural Products define as inactive a compound whose GI_50_ concentration is >10 *μ*M. The US National Cancer Institute (NCI, Bethesda, MD) also tests for a given compound 10 *μ*M as its higher potential growth inhibitory concentration. Consequently, if we refer to these criteria, among the studied compounds only compound** 6 **behaves actually as an inhibitor of cancer cell growth independently of the levels of sensitivity to proapoptotic stimuli of the various cancer cell lines under study.

On the other hand, compounds** 4** and** 5** showed a very similar pattern of growth inhibition with the most sensitive cell lines being MCF-7, SKMEL-28, and B16F10, while their growth inhibitory activity against the remaining three cell lines was about one magnitude weaker. This evidence seems to reflect the strong structural similarities among hepta- and octaprenylhydroquinones** 4** and** 5**, which just differ by one isoprene unit (Table 1). In addition, the data in Table 2 clearly indicate that FTAs and PHQs display quite distinct profiles in terms of* in vitro* cancer growth inhibitory activity, differing by one to two orders of magnitude in favor of the PHQs in all cell lines. Although additional structure-activity relationship (SAR) studies are necessary for accurate identification of the structural features required for the activity of compound** 6**, our finding suggests that the presence of a hydroxyl group on the chain of** 6** could be related to its enhanced growth inhibitory activity on cancer cells as compared to the nonhydroxylated** 4** and** 5**. This finding, however, contrasts with earlier findings indicating less efficiency of** 6** compared to** 4** and** 5** when tested against the chronic myelogenous leukemia (CML) cell line K562 [26]. This apparent discrepancy could mainly relate to the fact that Abed et al. [26] used one leukemia cell line growing in suspension, while we made use of six adherent cancer cell lines. As an example that we can cite among many others and thanks to the help provided by the NCI to characterize the mechanism of action of a novel fungal-related metabolite, that is, sphaeropsidin A, we observed that the leukemia cell lines from the NCI 60-cell line panel displayed markedly distinct profiles in terms of growth inhibition when compared, for example, to melanoma and kidney cancer cell lines [35]. The data from the current study clearly point to the fact that (i) compound** 6** (and to a lesser extent compounds** 4** and** 5**) markedly inhibits cancer cell growth and that (ii) the** 6**-induced growth inhibition in cancer cells is independent of the levels of resistance of the cancer cells (at least the models under study) to proapoptotic stimuli.

## 5. Conclusions

By comparing different metabolites isolated from sponges belonging to the genus* Sarcotragus*, our study evidenced higher* in vitro* growth inhibitory effects of PHQs on cancer cell lines, compared to FTAs. In addition, our results suggest, in spite of previous evidence reported in the literature, that compound** 6** is the most active among the tested PHQs, inducing growth inhibition in cancer cells independent of their levels of resistance to proapoptotic stimuli. Consequently,** 6 **clearly deserves further in-depth analyses of its anticancer mechanism of action, toward its possible use to treat metastatic cancer.

## Supplementary Material

The Supplementary Material includes NMR and mass spectra of compounds **1**–**6**, acquired using instruments and methods as described in the Materials and Methods section of the paper.

## Figures and Tables

**Table 1:** Structures of compounds **1**–**6**, natural sources, and sampling sites in Tunisia.

Structure	Sponge species	Site
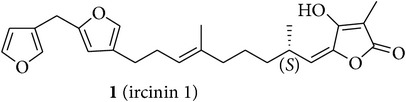	*Sarcotragus spinosulus *Schmidt, 1862	Tabarka
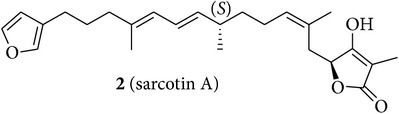	*Sarcotragus fasciculatus* (Pallas, 1766)	Monastir
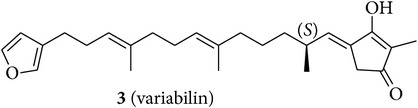	*Sarcotragus fasciculatus* (Pallas, 1766)	Monastir
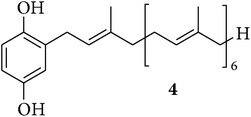	*Sarcotragus spinosulus *Schmidt, 1862	Monastir
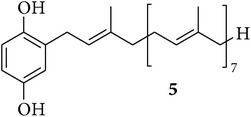	*Sarcotragus spinosulus *Schmidt, 1862	Tabarka
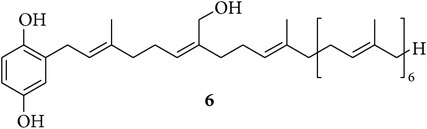	*Sarcotragus foetidus* Schmidt, 1862	Bizerte

**Table 2:** GI_50_ (*μ*M) values for compounds **1**–**6** against human and murine cancer cell lines.

Compound	Human					Murine	Mean ± SEM
	A549	Hs683	MCF-7	SKMEL-28	U373	B16F10	
**1**	>100	94	67	>100	>100	73	>89
**2**	>100	>100	>100	>100	>100	66	>94
**3**	>100	>100	>100	>100	>100	77	>96
**4**	23	27	3	3	15	1	12 ± 5
**5**	34	45	5	4	48	4	23 ± 9
**6**	4	5	4	1	5	1	3 ± 1

GI_50_: *in vitro* concentration (*μ*M) needed to inhibit cell population growth by 50% after 72 hours of cell culture with the compound.
