# Supplementary material for: In Vitro Growth Inhibitory Activities of Natural Products from Irciniid Sponges against Cancer Cells: A Comparative Study
Source: Biomed Res Int. 2016 Aug 11;2016:5318176. doi: 10.1155/2016/5318176 (PMC4997040; doi:10.1155/2016/5318176)
Supplement: Supplementary file 1 — The Supplementary Material includes NMR and mass spectra of compounds 1–6, acquired using instruments and methods as described in the Materials and Methods section of the paper. [file 5318176.f1.pdf]

Supplementary material

In vitro growth inhibitory activities of natural products from irciniid sponges against cancer cells: a comparative study

Yosr BenRedjem Romdhane,[1] Monia Elbour,[1] Marianna Carbone,[2] Maria Letizia Ciavatta,[2] Margherita Gavagnin,[2] Véronique Mathieu,[3] Florence Lefranc,[4] Leila Ktari,[1] Karim Ben Mustapha,[1] Abdellatif Boudabbous,[5] Robert Kiss,[3] and Ernesto Mollo[2]

[1]*Institut National des Sciences et Technologies de la Mer (INSTM), 2025 Salammbô, Tunis, Tunisia*

[2]*Consiglio Nazionale delle Ricerche (CNR), Istituto di Chimica Biomolecolare (ICB), 80078 Pozzuoli (Na), Italy*

[3]*Laboratoire de Cancérologie et de Toxicologie Expérimentale, Faculté de Pharmacie, Université Libre de Bruxelles (ULB), 1050 Brussels, Belgium*

[4]*Service de Neurochirurgie, Hôpital Erasme, ULB, 1070 Brussels, Belgium*

[5]*Laboratoire des Microorganismes et Biomolécules actives, Faculté des Sciences de Tunis, 2092 Tunis, Tunisia*

S1. ^{1}H NMR spectrum of ircinin (**1**) from *S. spinosulus* ($CDCl_3$, Bruker 400 MHz)

S2. ^{1}H NMR spectrum of ircinin (**1**) from *S. spinosulus* (CD_3OD, Bruker 600 MHz)

S3. HSQC spectrum of ircinin (**1**) from *S. spinosulus* (CD_3OD, Bruker 600 MHz)

S4. ESI MS spectrum of ircinin (**1**) from *S. spinosulus*

S5. ^{1}H NMR spectrum of sarcotin A (**2**) from *S. fasciculatus* ($CDCl_3$, Bruker 400 MHz)

S6. ^{1}H NMR spectrum of sarcotin A (**2**) from *S. fasciculatus* (CD_3OD, Bruker 400 MHz)

S7. ^{1}H-^{1}H COSY spectrum of sarcotin A (**2**) from *S. fasciculatus* ($CDCl_3$, Bruker 400 MHz)

S8. HSQC spectrum of sarcotin A (**2**) from *S. fasciculatus* ($CDCl_3$, Bruker 400 MHz)

S9. HMBC spectrum of sarcotin A (**2**) from *S. fasciculatus* ($CDCl_3$, J = 10Hz, Bruker 400 MHz)

S10. ESI-MS spectrum of sarcotin A (**2**) from *S. fasciculatus*

S11. ^{1}H NMR spectrum of variabilin (**3**) from *S. fasciculatus* ($CDCl_3$, Bruker 400 MHz)

S12. ^{1}H-^{1}H COSY spectrum of variabilin (**3**) from *S. fasciculatus* ($CDCl_3$, Bruker 400 MHz)

S13. HSQC spectrum of variabilin (**3**) from *S. fasciculatus* ($CDCl_3$, Bruker 400 MHz)

S14. HMBC spectrum of variabilin (**3**) from *S. fasciculatus* ($CDCl_3$, *J*= 10 Hz, Bruker 400 MHz)

S15. ESI MS spectrum of variabilin (**3**) from *S. fasciculatus*

S16. ^{1}H NMR spectrum of compound **4** from *S. spinosulus* ($CDCl_3$, Bruker 300 MHz)

S17. ^{13}C NMR spectrum of compound **4** from S. spinosulus ($CDCl_3$, Bruker 300 MHz)

S18. ESI MS spectrum of compound **4** from *S. spinosulus*

S19. ^{1}H NMR spectrum of compound **5** from *S. spinosulus* ($CDCl_3$, Bruker 400 MHz)

S20 ESI MS spectrum of compound **5** from *S. spinosulus*

S21. ^{1}H NMR spectrum of compound **6** from *S. foetidus* ($CDCl_3$, Bruker 400 MHz)

S22. HSQC spectrum of compound **6** from *S. foetidus* ($CDCl_3$, Bruker 400 MHz)

S23. ESI MS spectrum of compound **6** from *S. foetidus* sponge

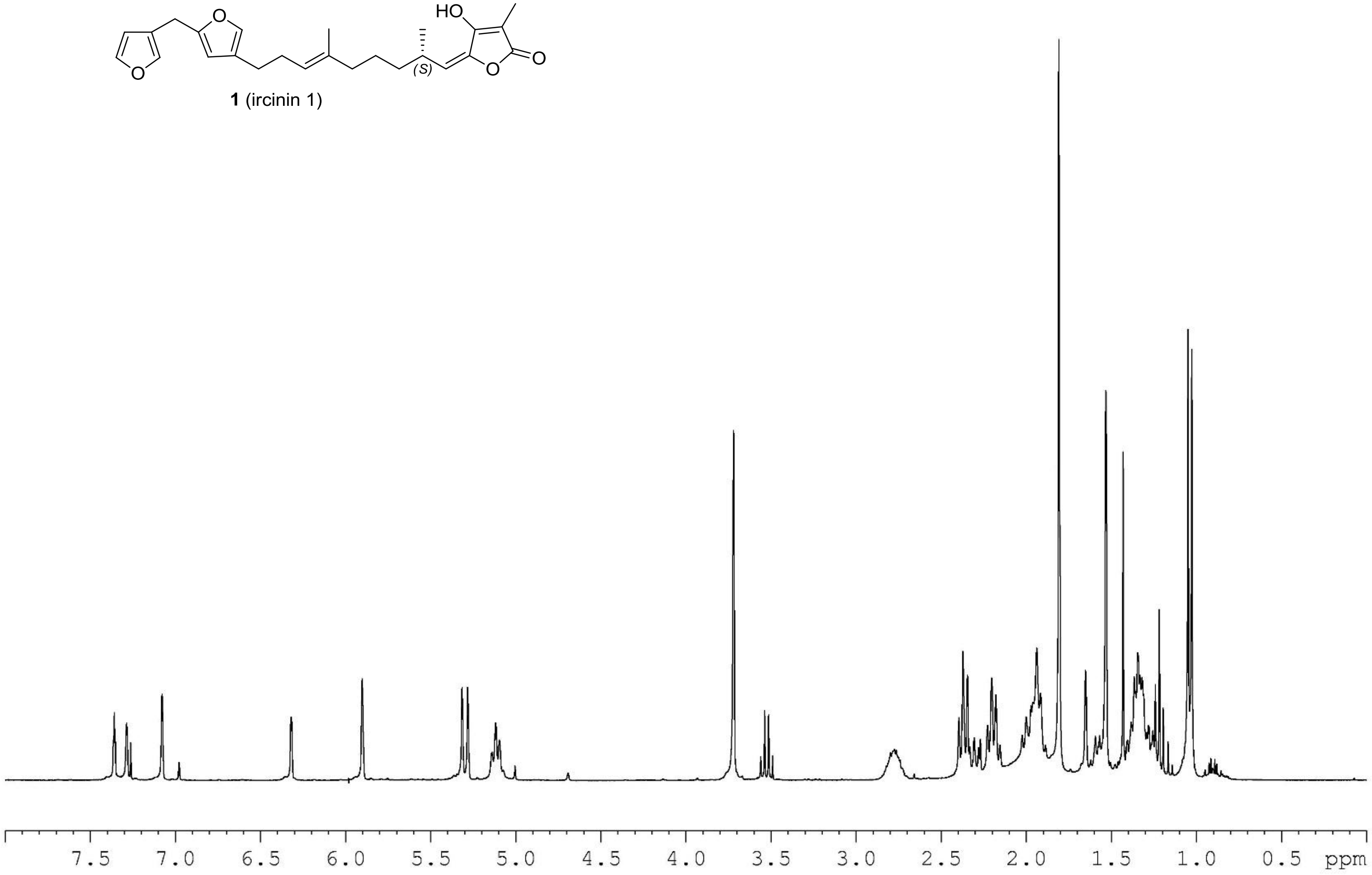

^{1}H NMR spectrum of ircinin (**1**) from *S. spinosulus* (CDCl**3**, Bruker 400 MHz)

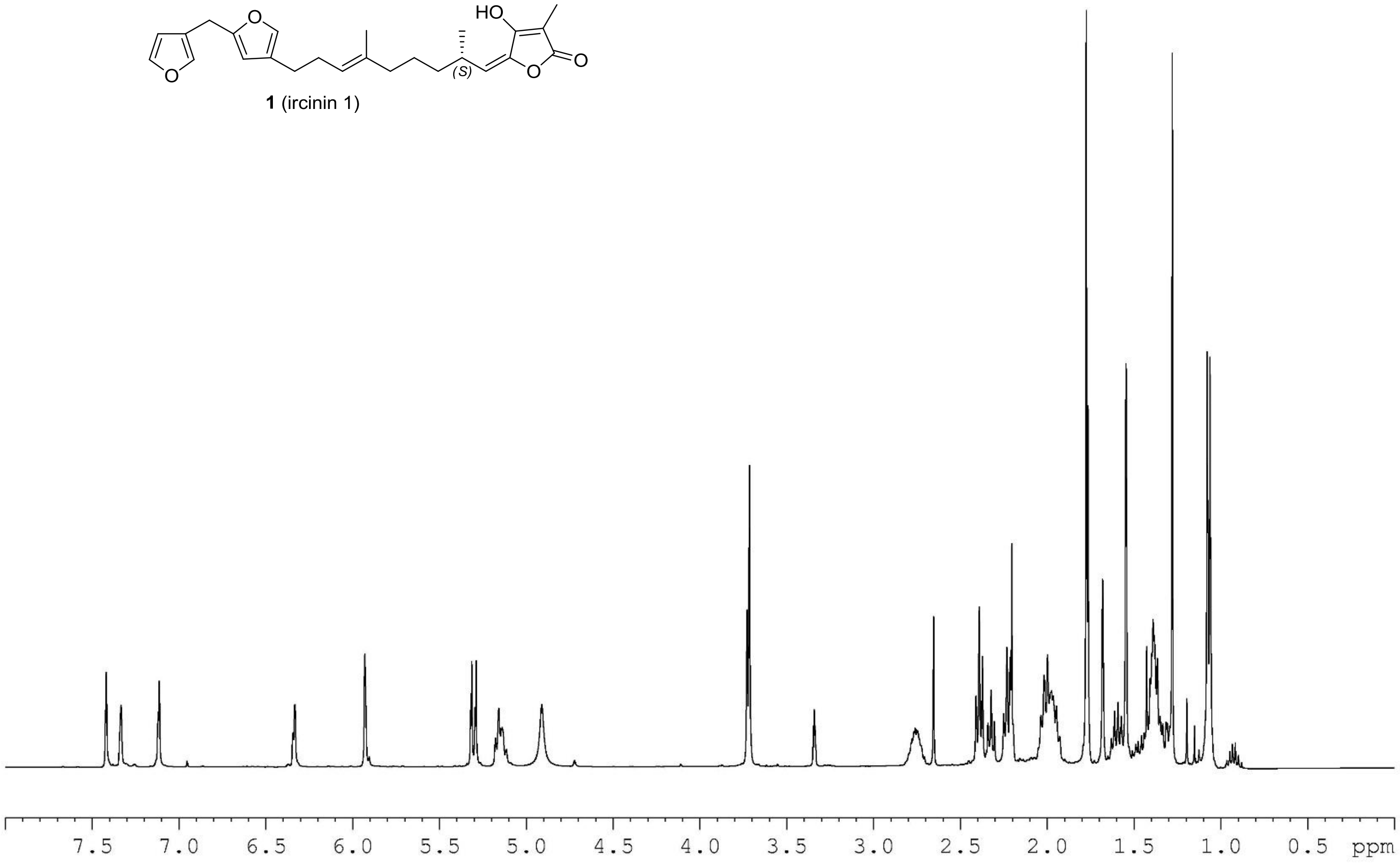

1 (ircinin 1)

^{1}H NMR spectrum of ircinin (**1**) from *S. spinosulus* (CD_3OD, Bruker 600 MHz)

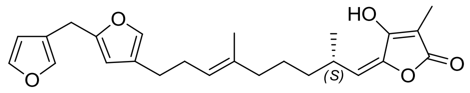

1 (ircinin 1)

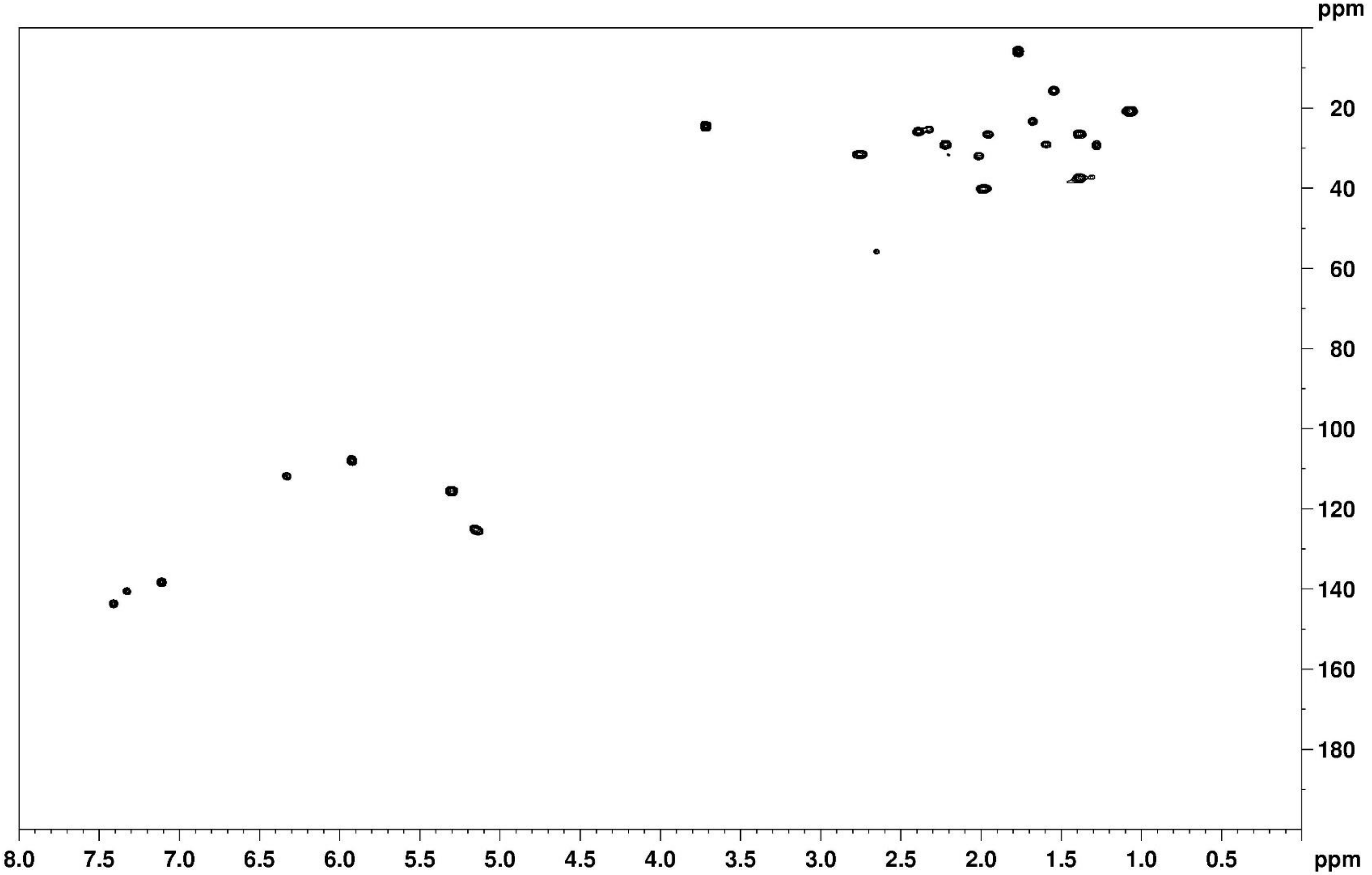

HSQC spectrum of ircinin (**1**) from *S. spinosulus* (CD_3OD, Bruker 600 MHz)

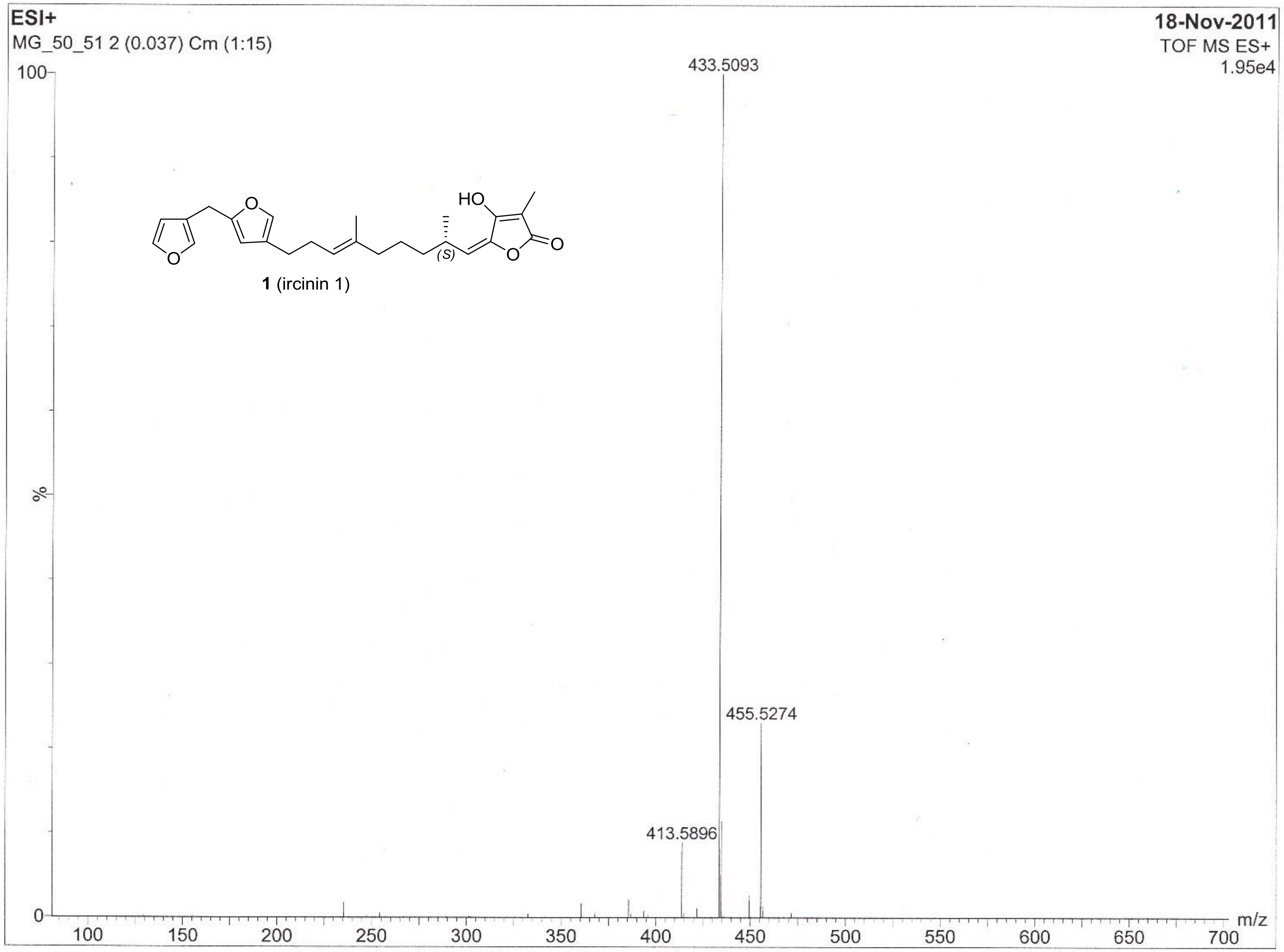

ESI MS spectrum of ircinin (**1**) from *S. spinosulus*

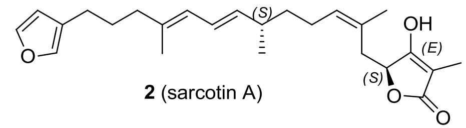

2 (sarcotin A)

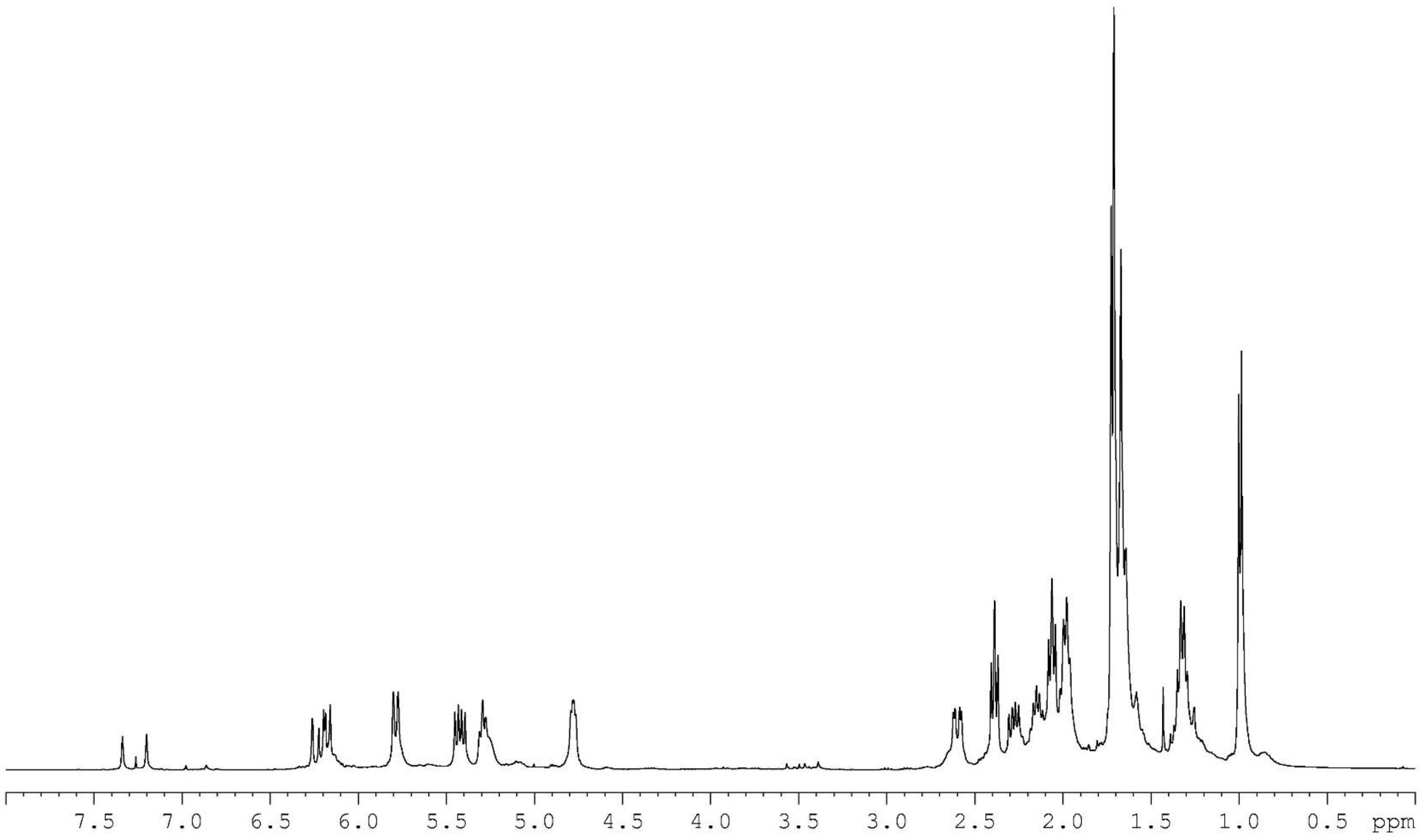

^{1}H NMR spectrum of sarcotin A (**2**) from *S. fasciculatus* ($CDCl_3$, Bruker 400 MHz)

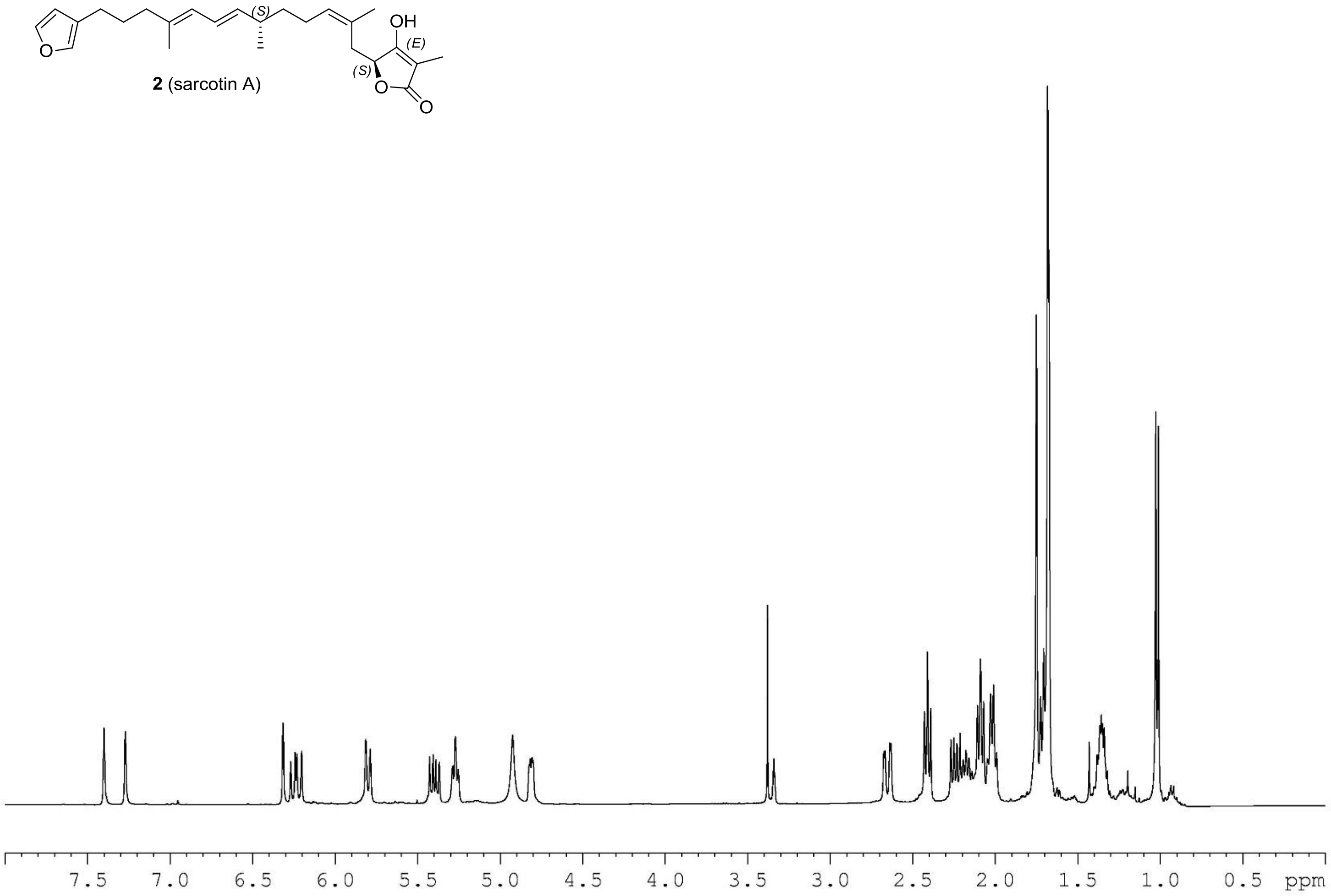

^{1}H NMR spectrum of sarcotin A (**2**) from *S. fasciculatus* (CD_3OD, Bruker 400 MHz)

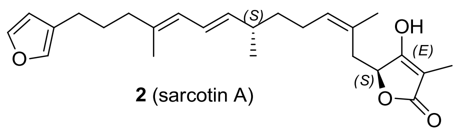

2 (sarcotin A)

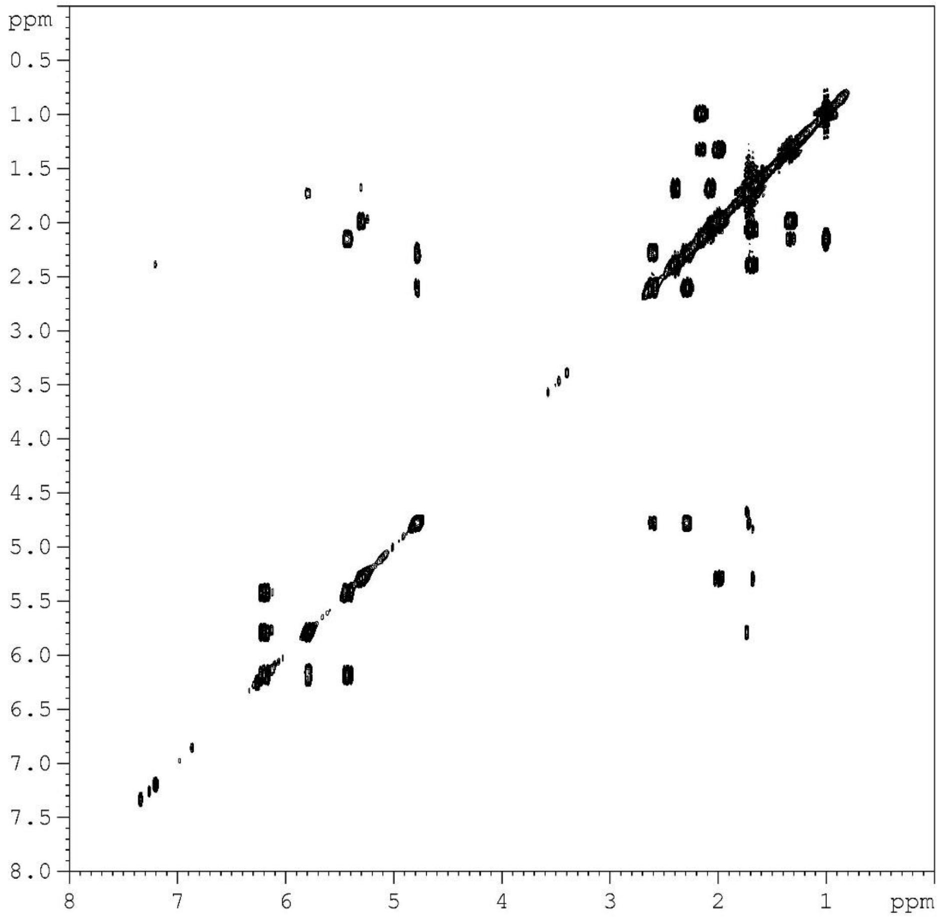

^1H-^1H COSY spectrum of sarcotin A (**2**) from *S. fasciculatus* ($CDCl_3$, Bruker 400 MHz)

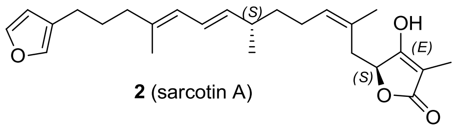

2 (sarcotin A)

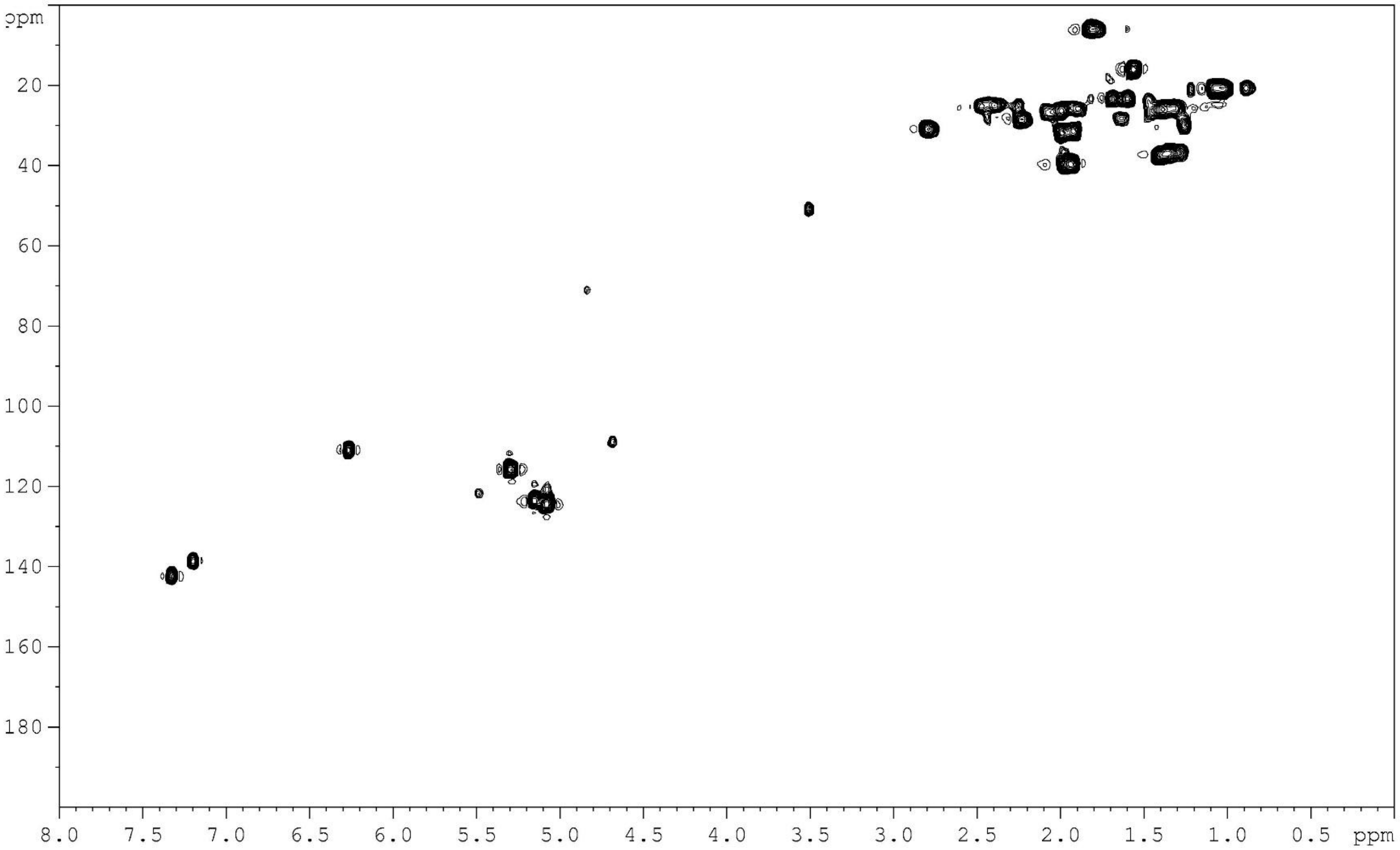

HSQC spectrum of sarcotin A (**2**) from *S. fasciculatus* ($CDCl_3$, Bruker 400 MHz)

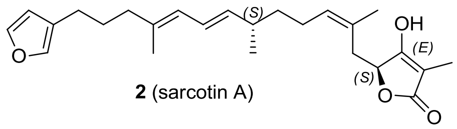

2 (sarcotin A)

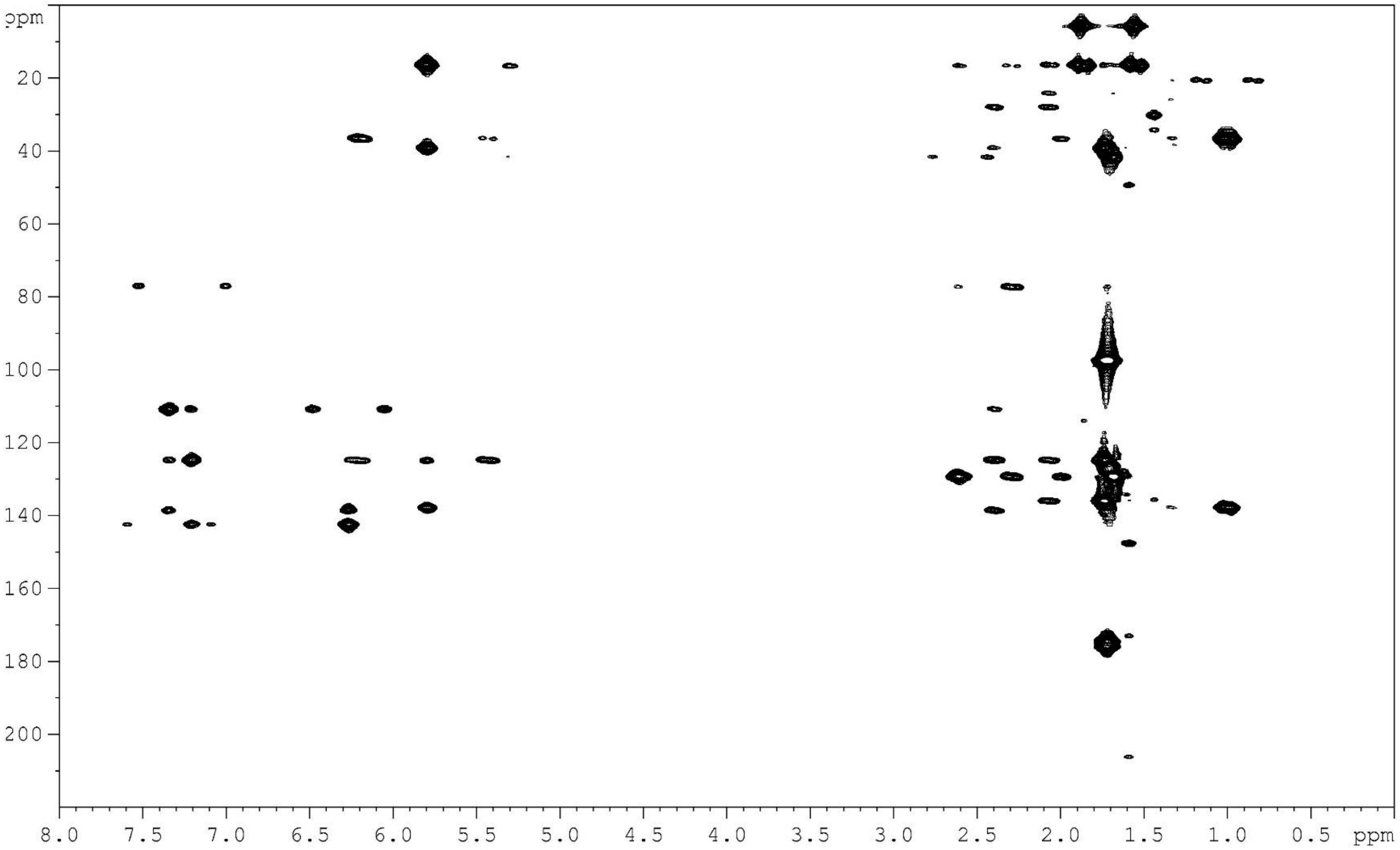

HMBC spectrum of sarcotin A (**2**) from *S. fasciculatus* ($CDCl_3$, *J* = 10Hz, Bruker 400 MHz)

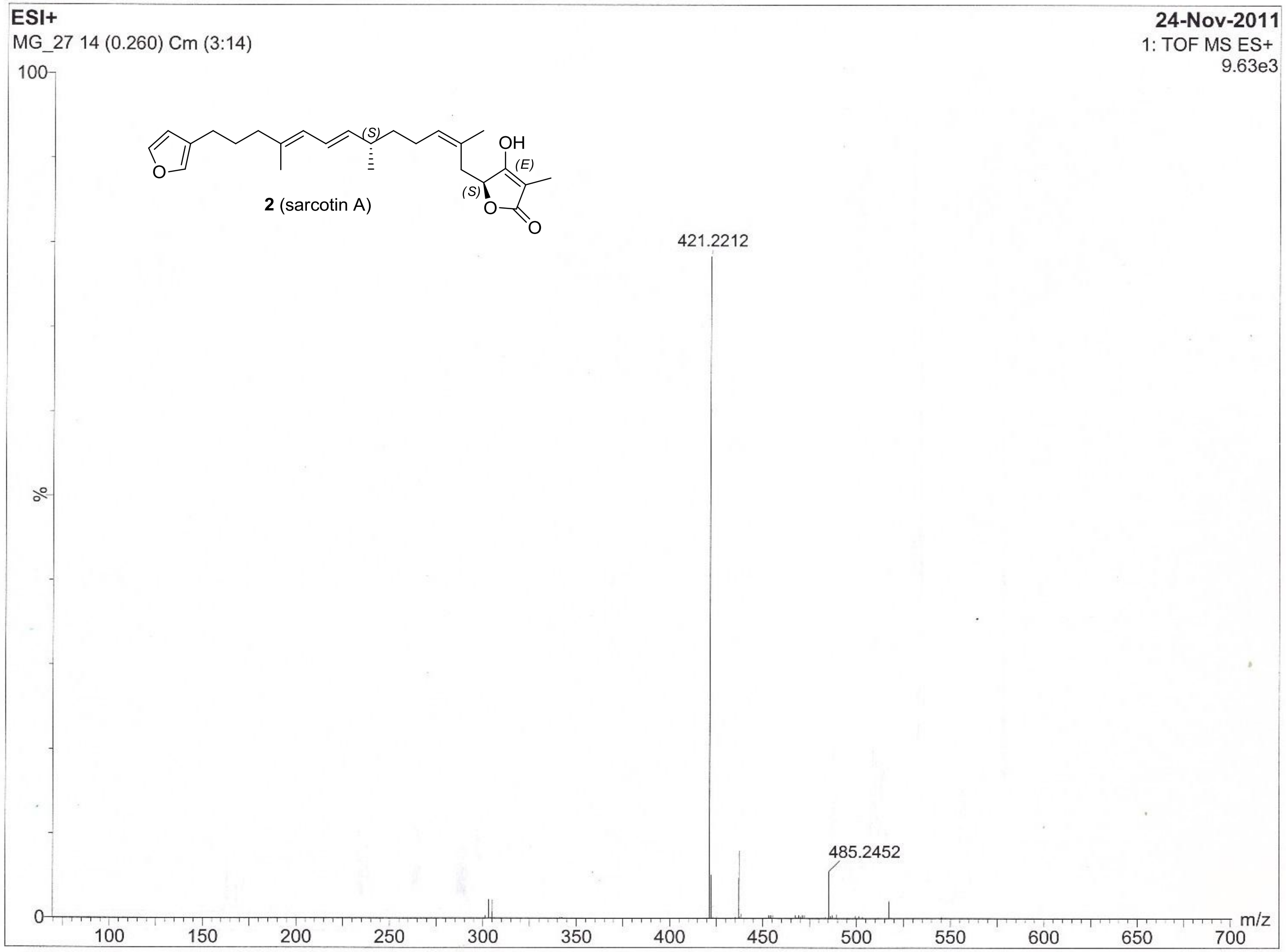

ESI-MS spectrum of sarcotin A (**2**) from *S. fasciculatus*

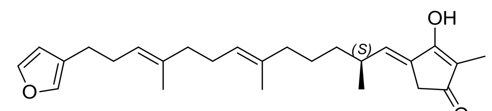

3 (variabilin)

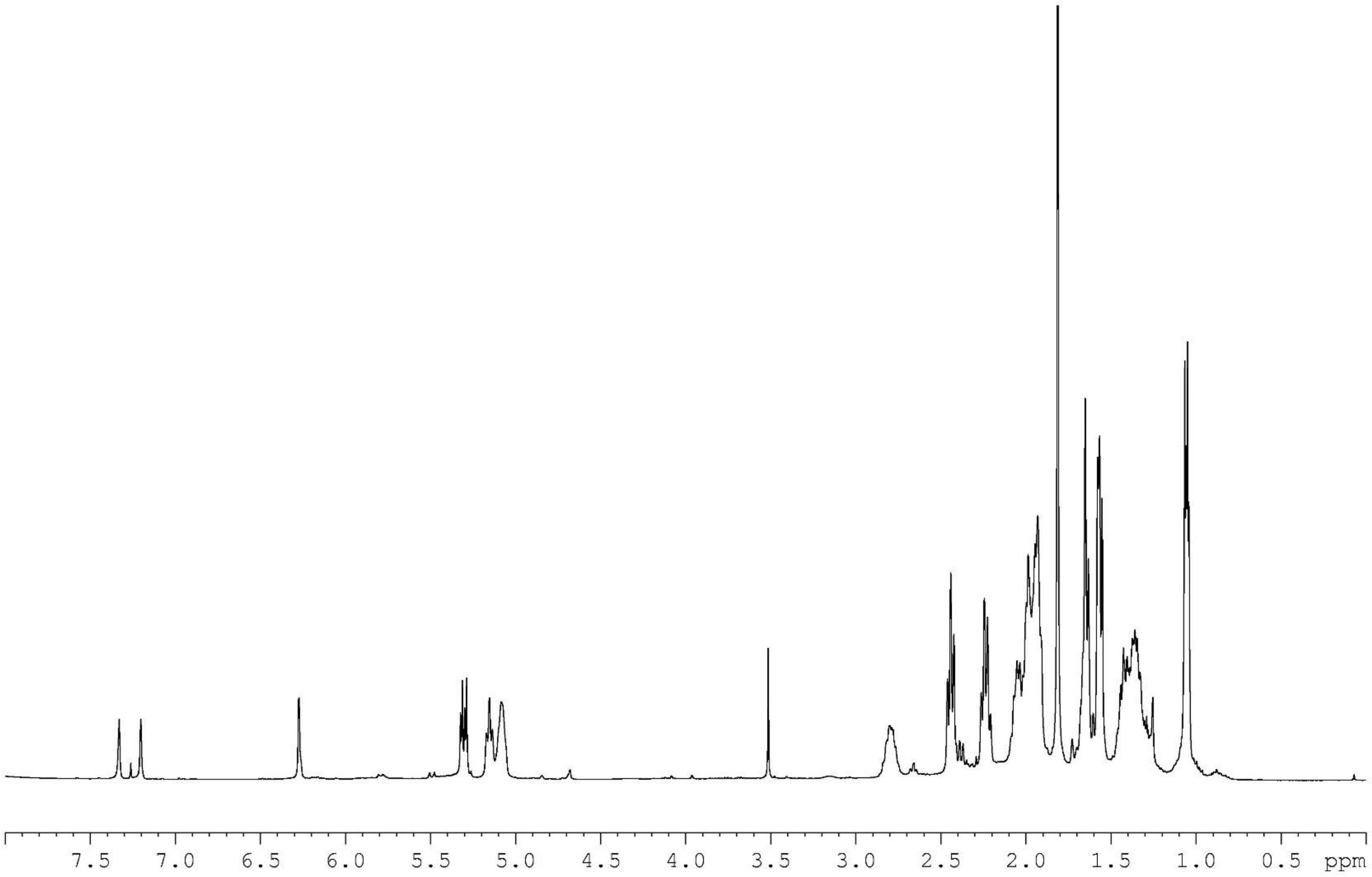

^{1}H NMR spectrum of variabilin (**3**) from *S. fasciculatus* ($CDCl_3$, Bruker 400 MHz)

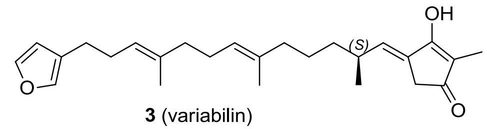

3 (variabilin)

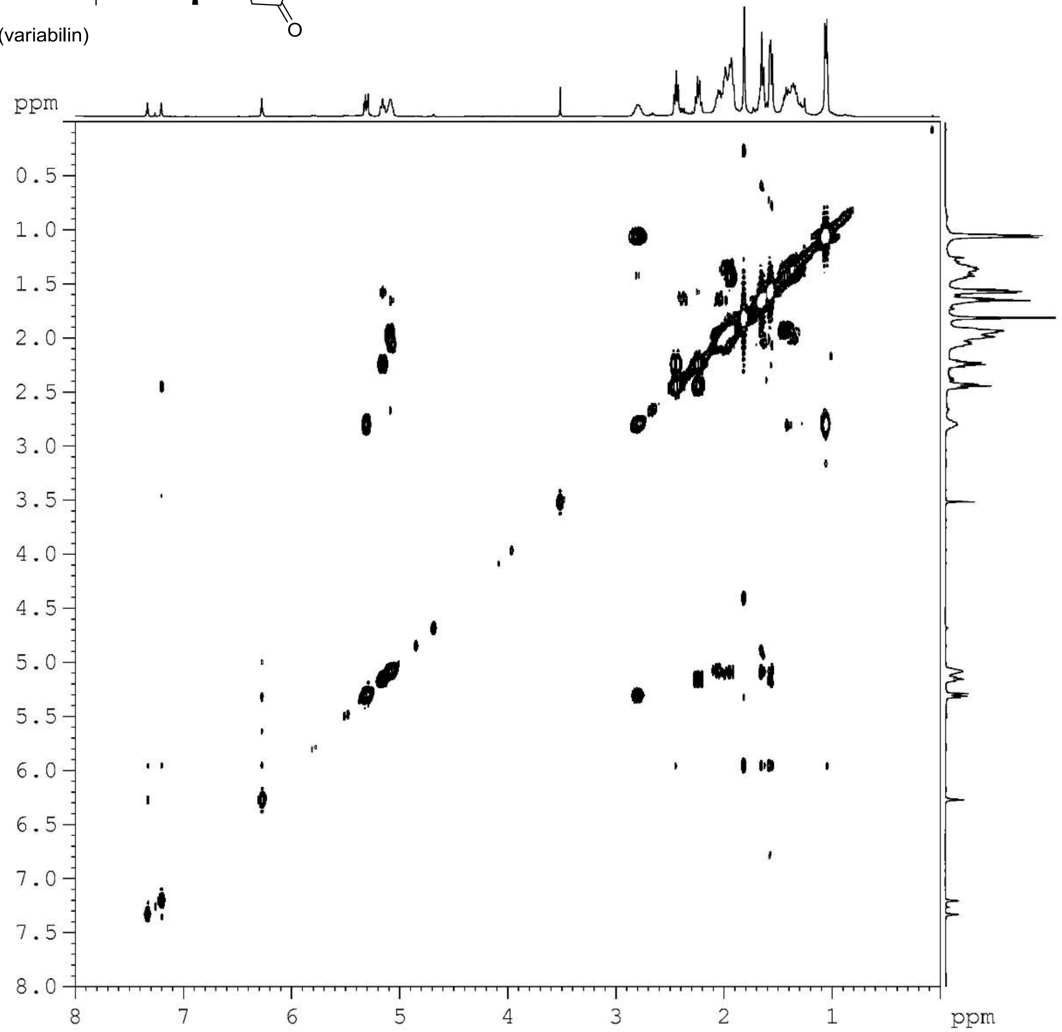

^{1}H-^{1}H COSY spectrum of variabilin (**3**) from *S. fasciculatus* ($CDCl_3$, Bruker 400 MHz)

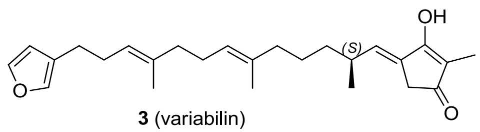

3 (variabilin)

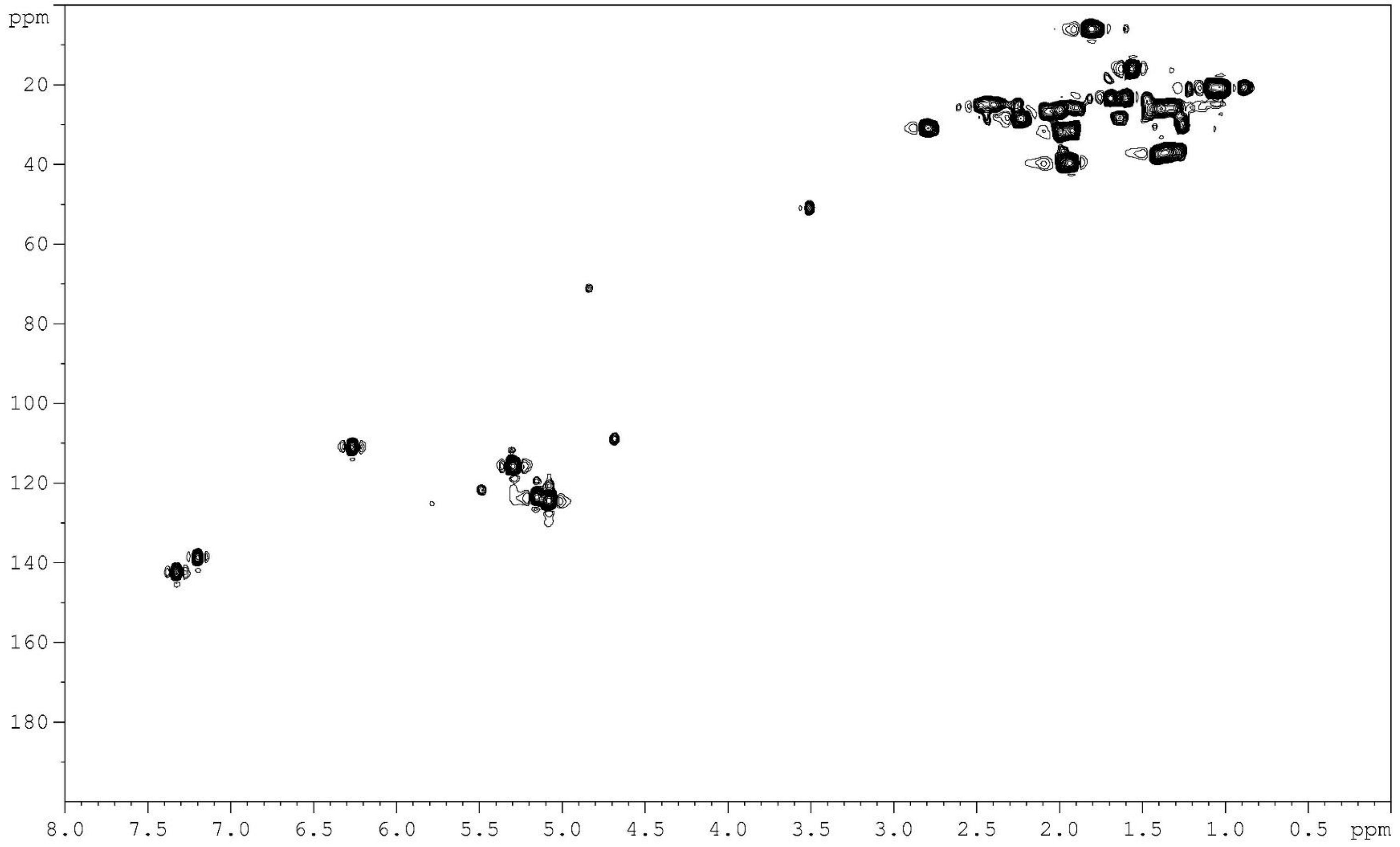

HSQC spectrum of variabilin (**3**) from *S. fasciculatus* ($CDCl_3$, Bruker 400 MHz)

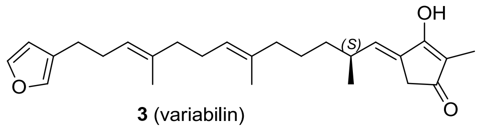

3 (variabilin)

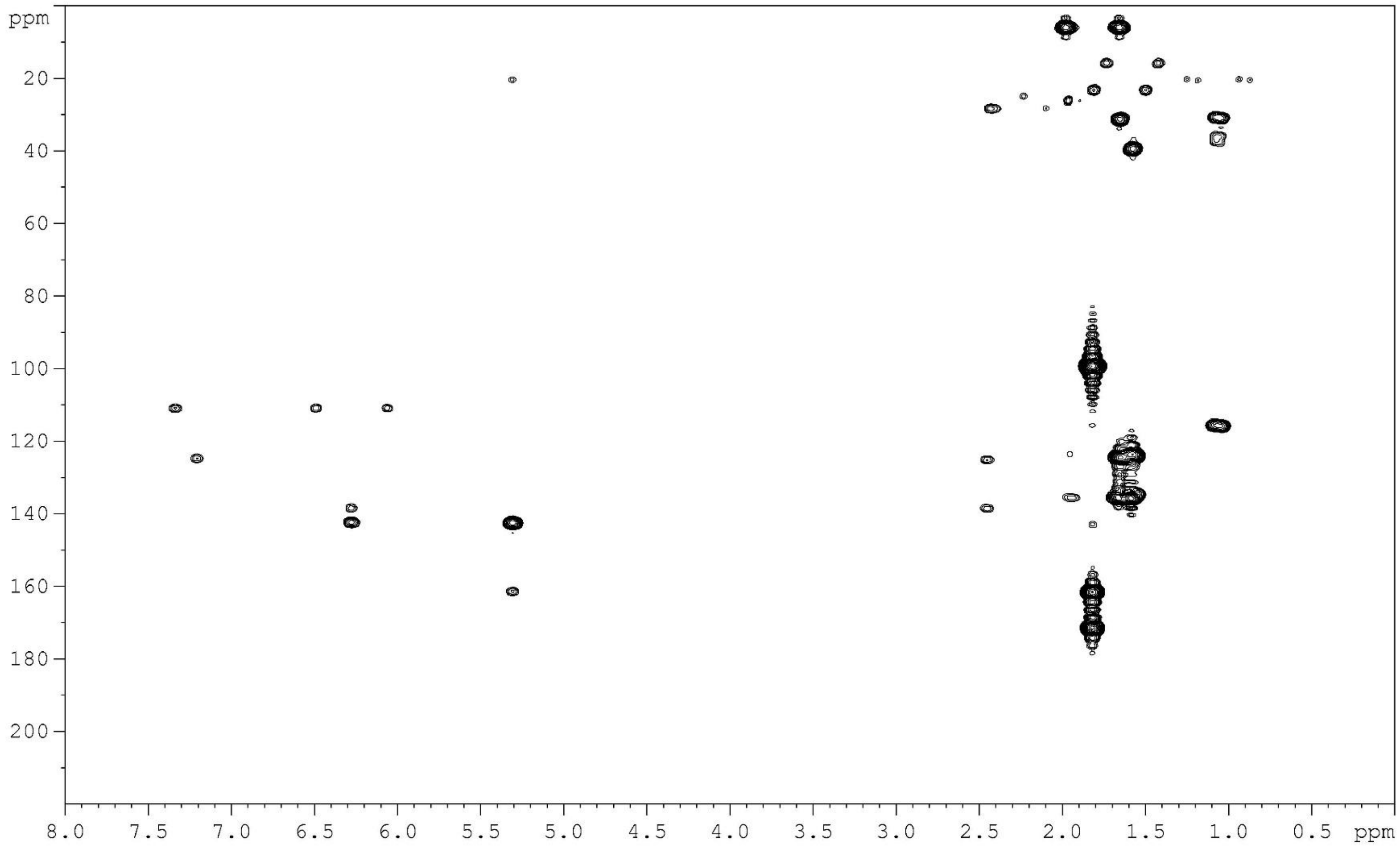

HMBC spectrum of variabilin (**3**) from *S. fasciculatus* ($CDCl_3$,*J*= 10 Hz) Bruker 400 MHz)

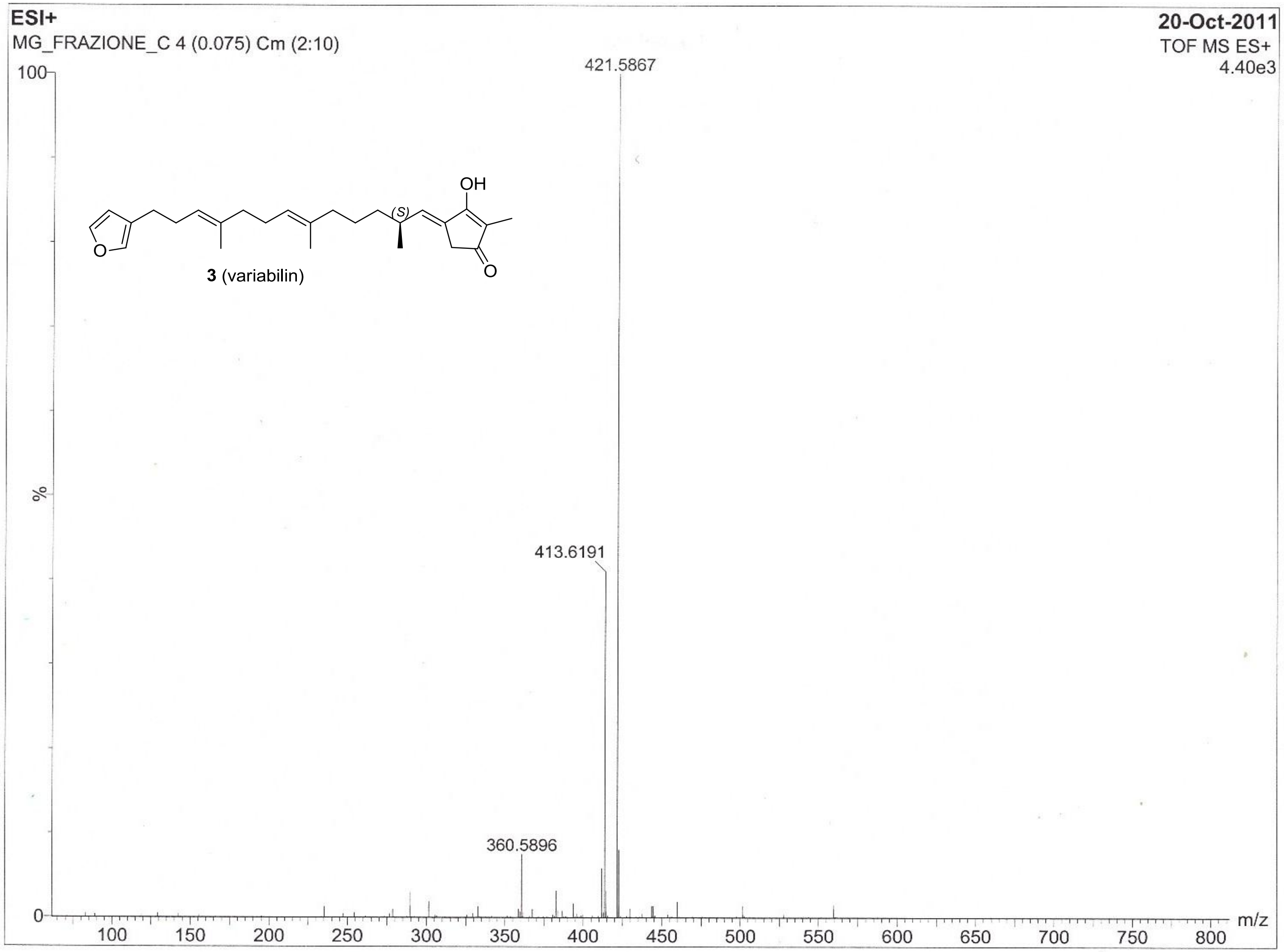

ESI MS spectrum of variabilin (**3**) from *S. fasciculatus*

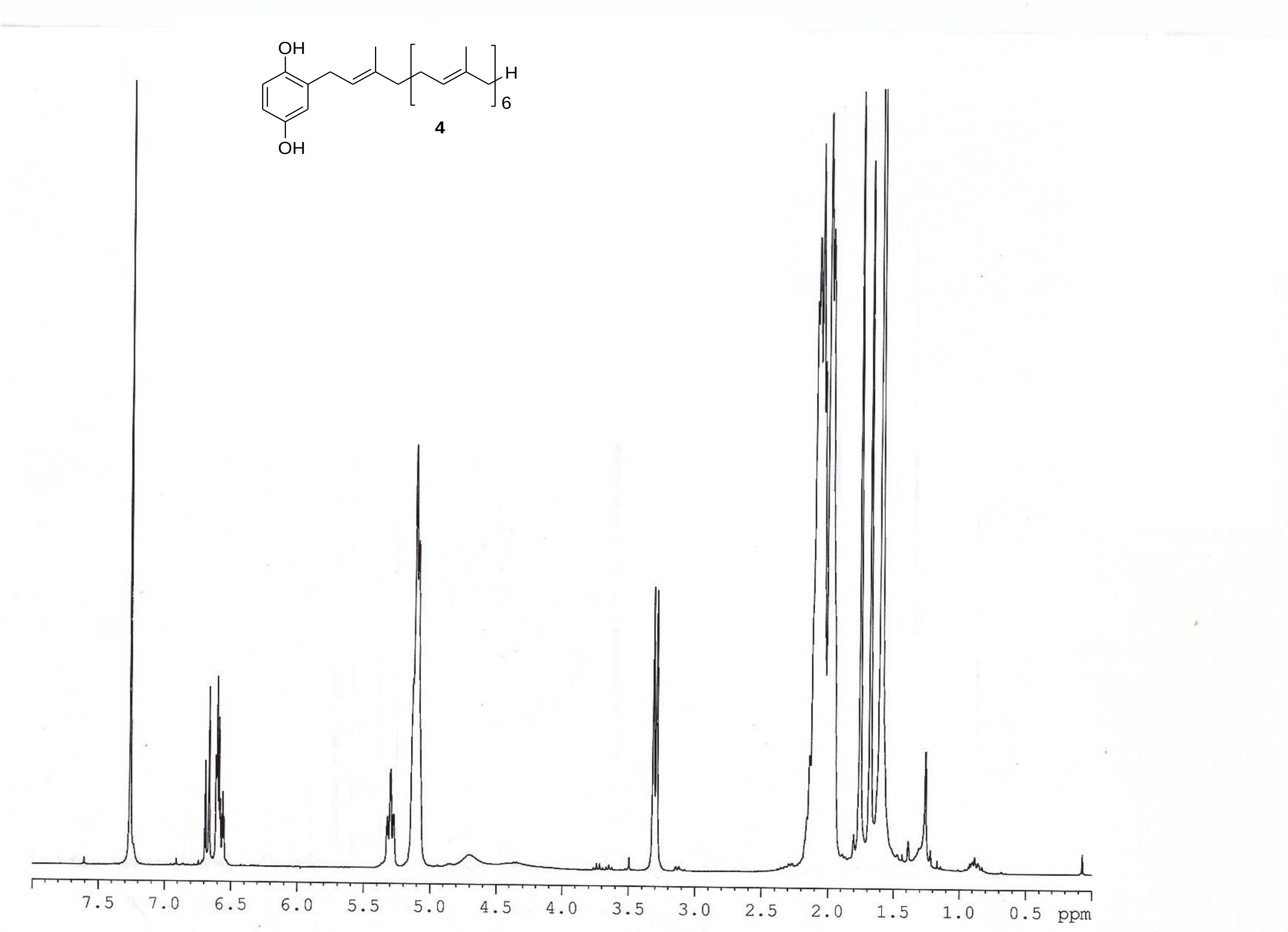

^{1}H NMR spectrum of compound **4** from *S. spinosulus* ($CDCl_3$, Bruker 300 MHz)

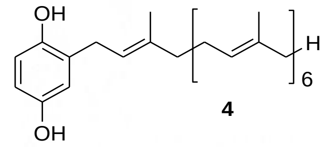

4

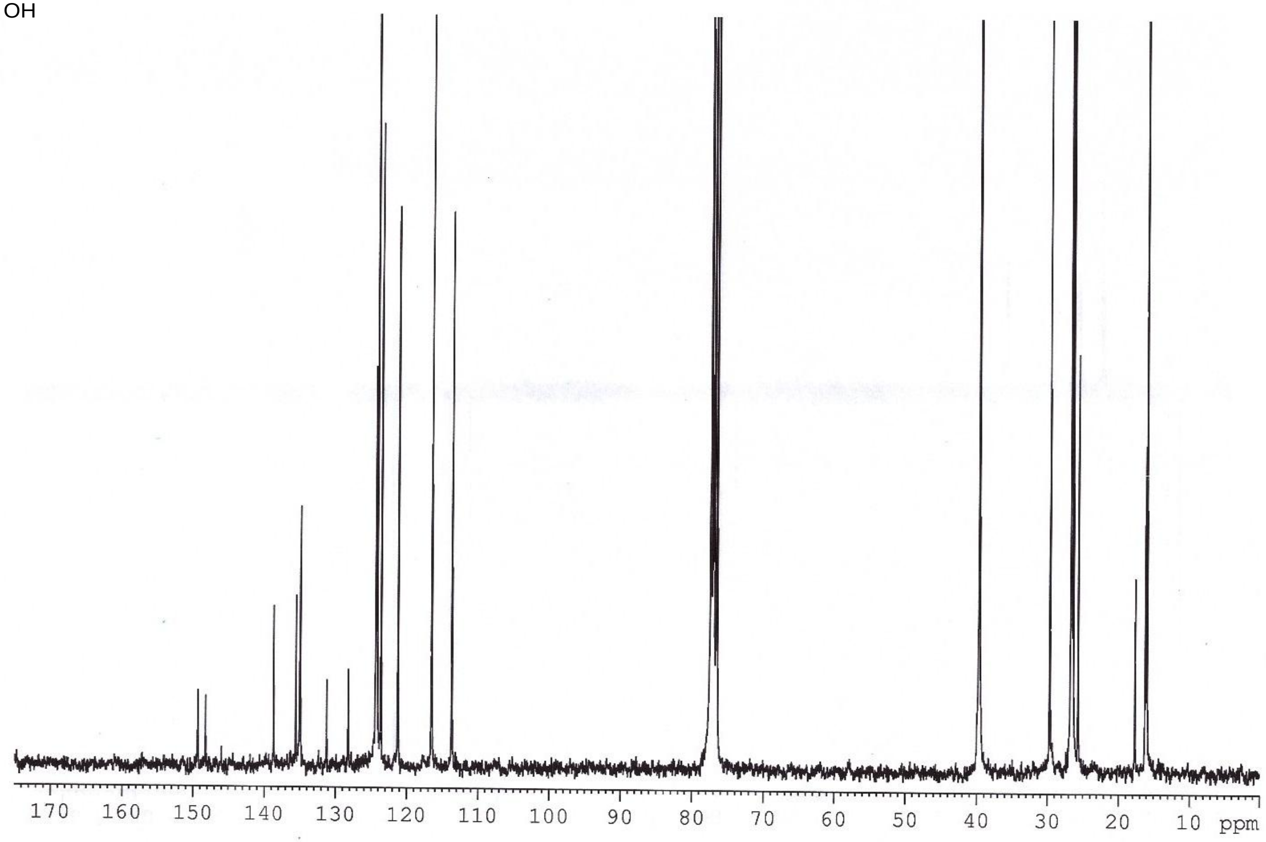

^{13}C NMR spectrum of compound **4** from *S. spinosulus* ($CDCl_3$, Bruker 300 MHz)

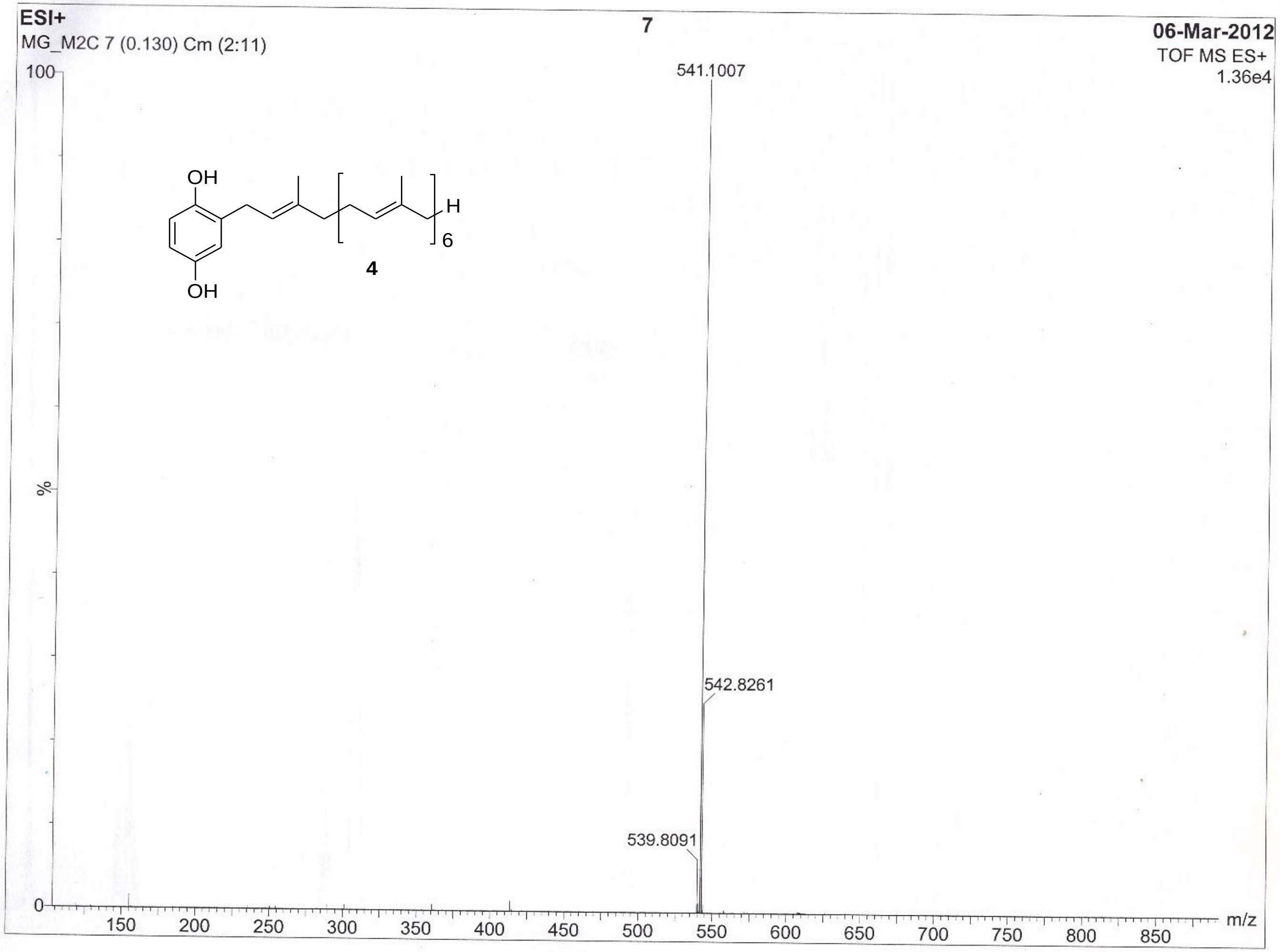

ESI MS spectrum of compound **4** from *S. spinosulus*

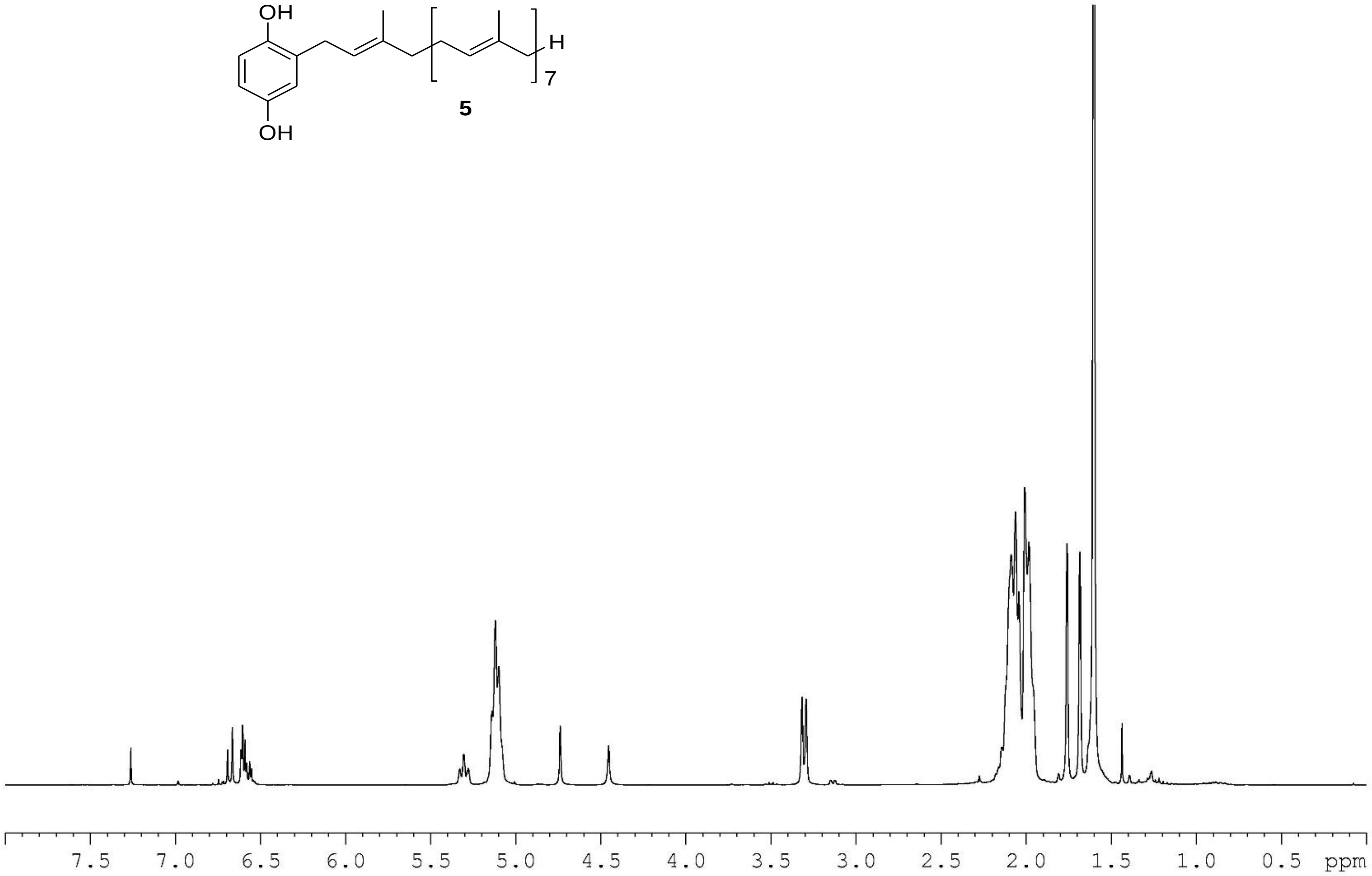

^{1}H NMR spectrum of compound **5** from *S. spinosulus* ($CDCl_3$, Bruker 400 MHz)

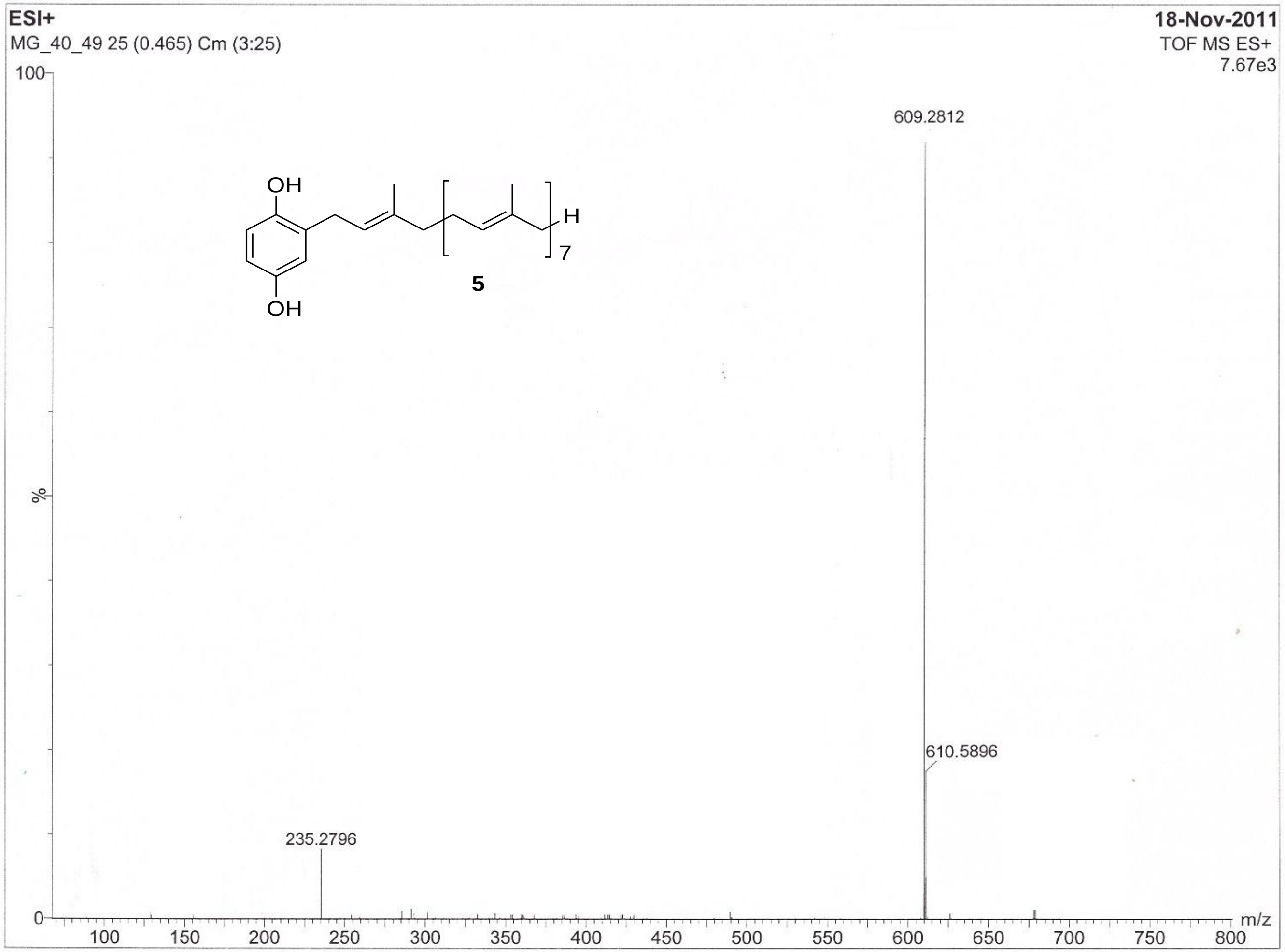

ESI MS spectrum of compound **5** from *S. spinosulus*

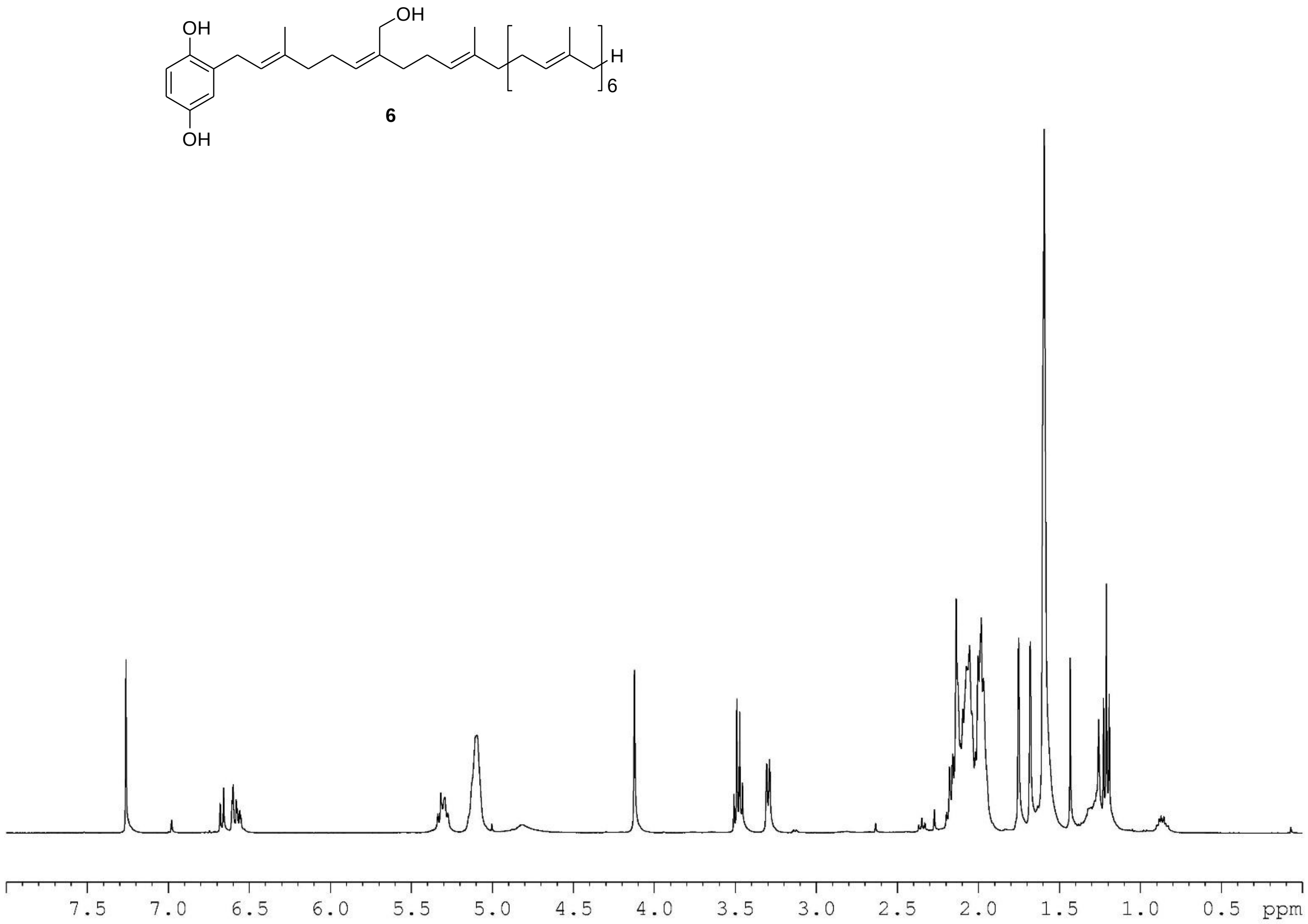

^{1}H NMR spectrum of compound **6** from *S. foetidus* ($CDCl_3$, Bruker 400 MHz)

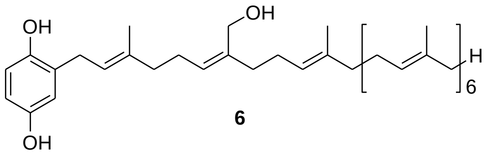

6

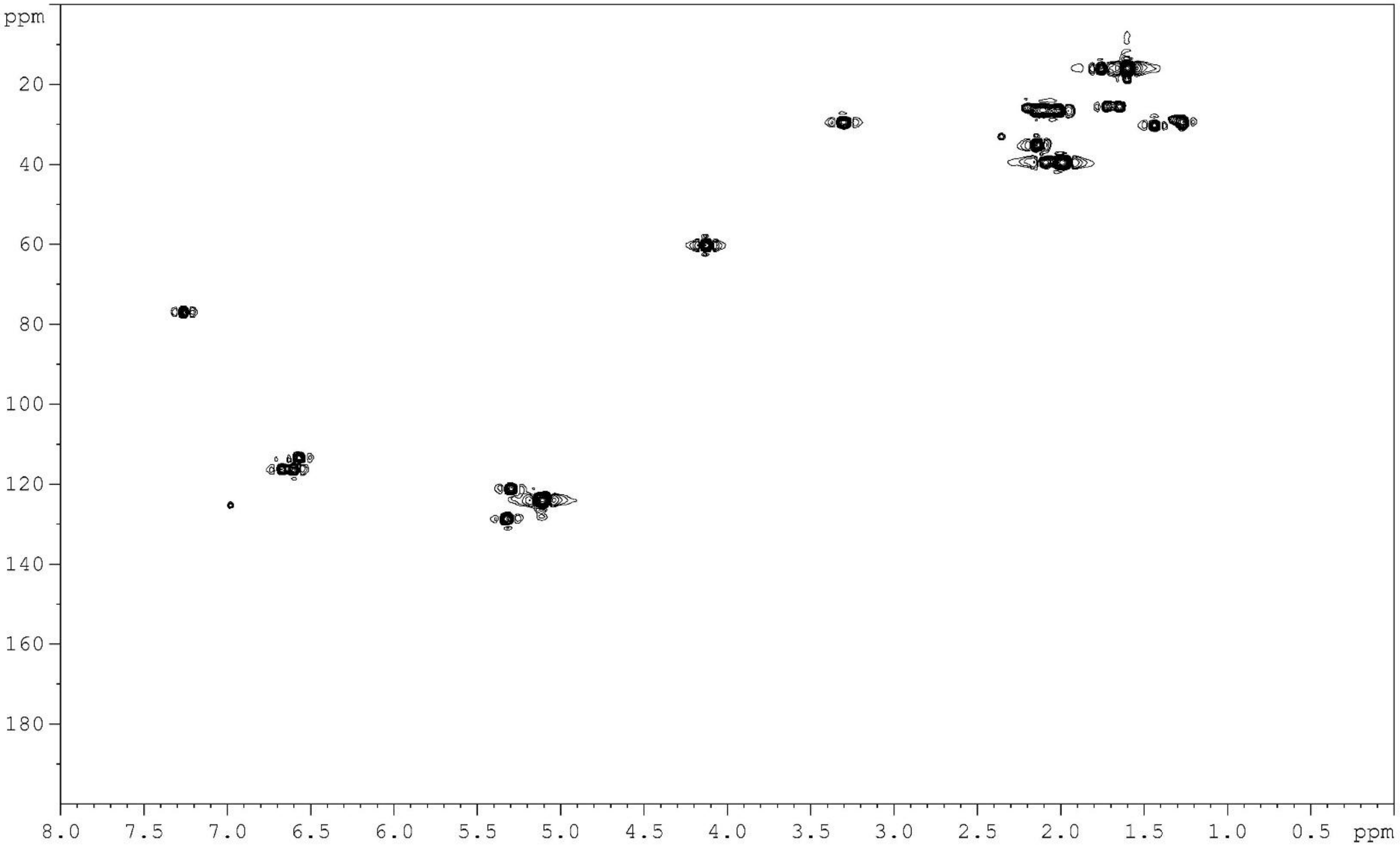

HSQC spectrum of compound **6** from *S. foetidus* ($CDCl_3$, Bruker 400 MHz)

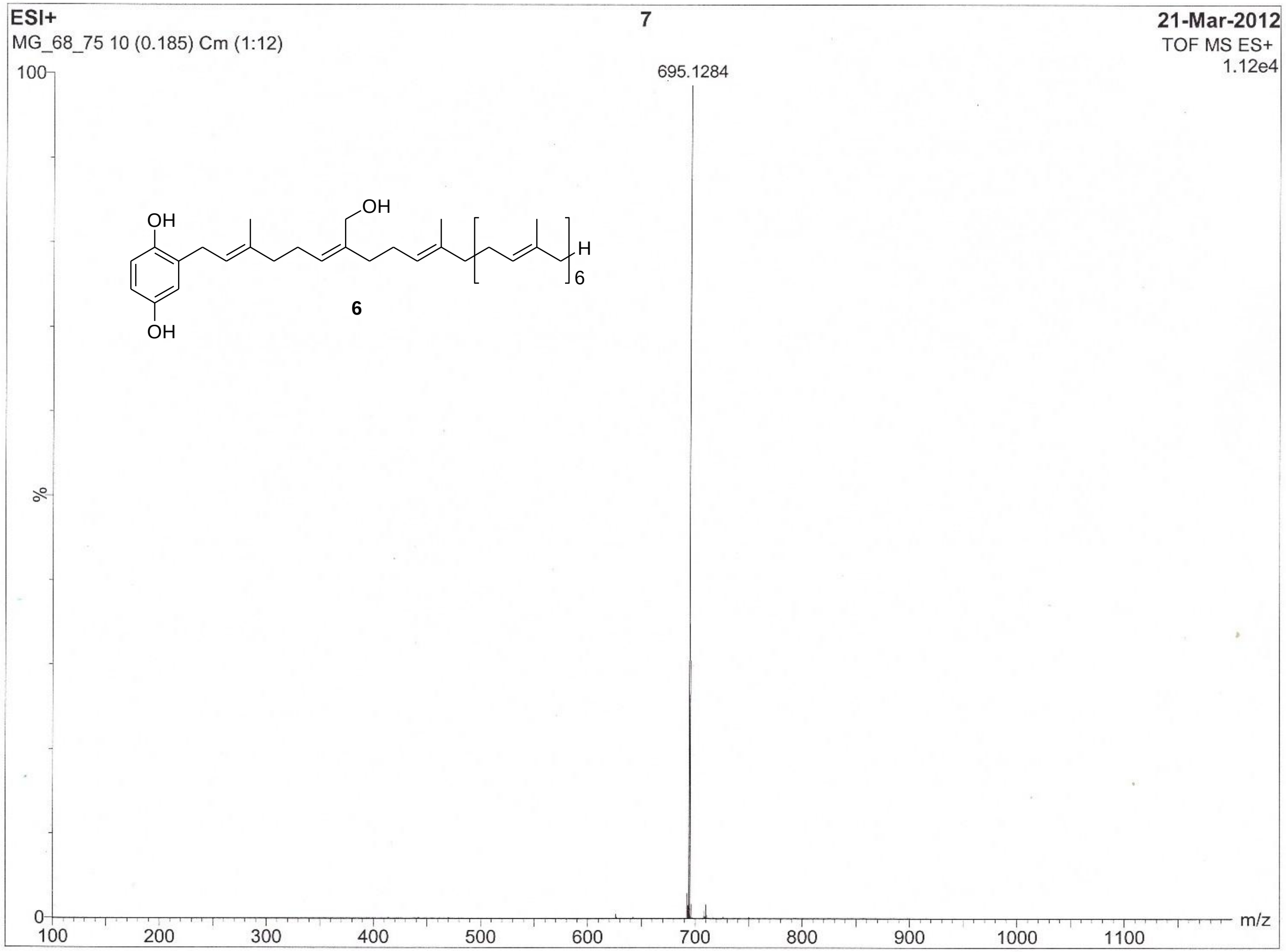

ESI MS spectrum of compound **6** *S. foetidus*
